# Dynamic Proteomic Characteristics and Network Integration Revealing Key Proteins for Two Kernel Tissue Developments in Popcorn

**DOI:** 10.1371/journal.pone.0143181

**Published:** 2015-11-20

**Authors:** Yongbin Dong, Qilei Wang, Long Zhang, Chunguang Du, Wenwei Xiong, Xinjian Chen, Fei Deng, Zhiyan Ma, Dahe Qiao, Chunhui Hu, Yangliu Ren, Yuling Li

**Affiliations:** 1 College of Agronomy, Henan Agricultural University, Collaborative Innovation Center of Henan Grain Crops, National Key Laboratory of Wheat and Maize Crop Science, 63 Nongye Rd, Zhengzhou, China; 2 Deptment of Biology and Molecular Biology, Montclair State University, Montclair, NJ 07043, United States of America; 3 College of Life Sciences, Henan Agricultural University, 63 Nongye Rd, Zhengzhou, China; Institute of Botany, Chinese Academy of Sciences, CHINA

## Abstract

The formation and development of maize kernel is a complex dynamic physiological and biochemical process that involves the temporal and spatial expression of many proteins and the regulation of metabolic pathways. In this study, the protein profiles of the endosperm and pericarp at three important developmental stages were analyzed by isobaric tags for relative and absolute quantification (iTRAQ) labeling coupled with LC-MS/MS in popcorn inbred N04. Comparative quantitative proteomic analyses among developmental stages and between tissues were performed, and the protein networks were integrated. A total of 6,876 proteins were identified, of which 1,396 were nonredundant. Specific proteins and different expression patterns were observed across developmental stages and tissues. The functional annotation of the identified proteins revealed the importance of metabolic and cellular processes, and binding and catalytic activities for the development of the tissues. The whole, endosperm-specific and pericarp-specific protein networks integrated 125, 9 and 77 proteins, respectively, which were involved in 54 KEGG pathways and reflected their complex metabolic interactions. Confirmation for the iTRAQ endosperm proteins by two-dimensional gel electrophoresis showed that 44.44% proteins were commonly found. However, the concordance between mRNA level and the protein abundance varied across different proteins, stages, tissues and inbred lines, according to the gene cloning and expression analyses of four relevant proteins with important functions and different expression levels. But the result by western blot showed their same expression tendency for the four proteins as by iTRAQ. These results could provide new insights into the developmental mechanisms of endosperm and pericarp, and grain formation in maize.

## Introduction

Maize (*Zea mays* L.), an important cereal grass, is widely grown as food, livestock fodder, and raw material for energy and other industrial uses in worldwide. Therefore, the characteristics of grain development have been widely studied to improve yield and nutritional quality [1–8]. Maize grain consists of the triploid endosperm, diploid embryo and maternal pericarp [9]. These three tissues develop coordinately and make up 70–90%, 10–12% and 5–6% of the grain dry weight, respectively [10–12].

The whole endosperm development in maize can be divided into five stages, the coenocytic phase, cellularization, differentiation, reserve synthesis and maturation [4, 13]. The first stage begins immediately after pollination, during which the triploid endosperm nucleus undergoes synchronous division in the absence of cell division. The cellularization around the nuclei begins to form a cell wall until about 3–6 days after pollination (DAP). Next, cell division and endoreduplication in the endosperm occur within 10–15 DAP. The cells differentiate into distinct tissue types, including transfer cells, aleurone, the embryo-surrounding region and starchy endosperm [14]. After about 12 DAP, starch and storage proteins begin to accumulate, which involve several metabolic processes [4, 5, 13, 15–17]. The quantity of dry-matter in the endosperm tissue is mainly synthesized at about 12–35 DAP [4, 15]. At about 16 DAP, the starchy endosperm cells begin to undergo programmed cell death (PCD) [14]. Cell division and grain filling are the important biological processes in grain development, which determine the dry matter content of mature grains and provide rich nutrients for the developing seed and germinating embryo [14, 18–20]. After about 35 DAP, the dry-matter accumulation decrease in the endosperm, and the kernel start to dehydrate quickly [4, 5].

The maternal pericarp tissue develops from the ovary wall in maize, which is mainly composed of carbohydrates, particularly corn fiber or cellulose. At about 10 DAP, the central cells in the pericarp start collapsing gradually from the crown, spreading down the sides of the kernel. The cells of inner pericarp collapse to accommodate the expanding endosperm at about 20 DAP. However, outer cells elongate with significant thickening cell walls to form a protective coat on the mature kernel [21]. In addition to being protection for the embryo and endosperm, pericarp thickness plays important roles in determining grain quality, especially for popcorn and sweet corn varieties. In fact, pericarp thickness and extensibility could restrict grain volume development during grain filling, which finally affects the weight of mature grains [22–23]. The differences in pericarp overall thickness were due to the width of individual pericarp cell walls and the number of pericarp cell layers [24]. During the grain maturity stage, the drying rates were associated with pericarp thickness and permeability, not to metabolic processes within the mature grain [25–27].

Grain formation and development is a complicated physiological and biochemical process, which involves the temporal and spatial expression of many genes and the regulation of metabolic pathways [28]. Several complex and inter-related metabolic processes are involved in maintaining normal metabolism during maize grain development [3, 13, 15–17, 29]. With the development of biological technologies, gene and protein expression profiles can be identified [30–31]. Accordingly, a metabolic network reconstruction could uncover the molecular mechanisms of a particular organism, which would help unravel the systemic biology of the metabolic pathways [32–33]. This could also contribute to the identification of genes or proteins that play important roles in the biological and chemical processes involved in the developmental process [19]. By constructing and analyzing cDNA libraries, endosperm development was thoroughly characterized and some tissue-specific genes were obtained [1, 2, 5, 7, 8, 30]. At least 5,000 different genes could be expressed during endosperm development. Moreover, the expression patterns of many imprinted genes, which were recognized by the deep sequencing of mRNAs from maize endosperm, were revealed [34–35].

However, a direct relationship between mRNA levels and protein abundance is difficult to be assessed [31, 36]. From mRNA to protein, some factors influence the coordination between mRNA levels and protein abundance, such as posttranscriptional regulation and posttranslational modification, as well as differential protein and mRNA degradation rates [37–39]. The protein level might reflect the real function of genes in certain tissues or times, which would help uncover their functions. In recent years, some research techniques for comprehensive proteomic analyses have been improved greatly. Although two-dimensional gel electrophoresis (2DE) is an established technique for protein analysis, it has several disadvantages, including being less efficient in identifying low-abundant, extreme isoelectric and hydrophobic proteins, and poor reproducibility, which limits its extensive application [40–42]. The combination of high performance liquid chromatography (HPLC) and mass spectrometry (MS), and liquid chromatography coupled with tandem mass spectrometry (LC-MS/MS), can greatly improve the sensitivity and specificity of protein identification. Some quantitative proteomics techniques, such as isobaric tags for relative and absolute quantification (iTRAQ) labeling [43], two-dimensional difference gel electrophoresis (2D-DIGE) [44], isotope-coded affinity tags (ICATs) [28] and label-free techniques [45], have been widely applied in plants.

The major advantages of iTRAQ are its high sensitivity and accuracy in protein identification, and the simultaneous quantification [46]. Therefore, this technique has been used to obtain quantitative proteomic data for different organs and tissues in several plants. The protein profile of *Arabidopsis* roots under conditions of Fe deficiency and excess Zinc were comprehensively analyzed using iTRAQ [47–48]. Owiti et al. [49] analyzed the expression profiles of soluble and non-soluble cassava root proteins during a 96-h post-harvest time course, from which a comprehensive proteome map of the cassava root was established and the regulated proteins were identified. In maize, Silva-Sanchez et al. [50] performed a quantitative proteomics analysis of the maize *miniature1* (*mn1*) mutant and its wild-type *Mn1*. Roy et al. [51] identified 64 differentially expressed protein spots and 140 proteins from the differential proteome of wild-type and mutant pericarps (*unstable factor for orange1*-1, *Ufo1*) using 2D-DIGE and iTRAQ, respectively. Until now, limited researches have been reported on the proteomic comparison between different kernel tissues in maize. The special protein network integration has not been reported yet [38].

Popcorn is a special kind of corn type, which was used as a popular snack around the world because of its tasty, convenient and nutritious properties. Unlike normal maize, popcorn kernels are much more flint due to hard pericarp and endosperm, and are capable of withstanding a higher internal pressure to obtain its maximum popping volume. In fact, the special characteristics in hard pericarp and endosperm for popcorn kernels are also very important in normal maize varieties [52]. In this research, the protein profiles of endosperm and pericarp at three key developmental stages in popcorn inbred N04 were identified using iTRAQ labeling coupled with LC-MS/MS. Accordingly, comparative quantitative proteomic analyses among developmental stages, and between the two tissues were performed using bioinformatics, and the protein networks were integrated. Our main objectives were to reveal key proteins in controlling grain development by uncovering the dynamic protein expression profiles and the comprehensive differences between two tissues, and to construct protein networks that revealed the relationships among detected proteins. These results would provide new insights into the developmental mechanisms of the endosperm and pericarp, and grain formation in popcorn.

## Materials and Methods

### Plant materials and sample collection

A popcorn inbred line N04 with a small-sized grain were planted at the Scientific Research and Education Center of Henan Agricultural University in Zhengzhou, Henan, China, in 2011. 30 rows of 4 m were planted with 0.67 m spacing between rows. Standard cultivation management practices were performed. All plants were self- or sib-pollinated when more than 80% of silks appeared.

Ears were harvested once every 2 DAP or 3 DAP. To increase the uniformity of the isolated grains, the upper half and about one-sixth from the bottom of the ears were cut and discarded. Grains were isolated from the remaining parts of the ears. Samples were collected from at least six ears and pooled at each time point for three biological replications. The two grain component parts, endosperm and pericarp, were manually separated after 10 DAP. Some of the collected samples were frozen in liquid nitrogen immediately and stored at –80°C, while others were used to measure fresh and dry weight.

### Light microscopy and scanning electron microscopy

For light microscopy analysis, the kernels were prepared and observed as described by Takacs et al. [53], with some modifications. Briefly, kernels were fixed in formaldehyde-acetic acid solution that contained 10% formalin, 5% acetic acid and 50% ethanol. The fixed kernels were dehydrated in a graded ethanol series (50, 70, 85, 95 and 100% ethanol, each for 30 min), then cleared with xylene and paraffin wax infiltration. After clearing, the samples were embedded and sectioned at 8–10 μm thickness under a Leica RM2235 Biocut. The sections were stained with Safranin O and Fast Green, and photographed with a Leica DM4000B microscope. Cell counts were measured by ImageJ analysis software [54–55].

For the scanning electron microscopy (SEM) analysis, the kernels were prepared and observed as described by Lending and Larkins [56]. Dry mature kernels were rifted with a blade along the longitudinal axis and spray coated with gold in an E-100 ion sputter. Gold-coated samples were then examined using a S3400N microscope at 5 kV.

### Protein isolation and digestion

For each sample, both endosperm and pericarp tissues (~0.5 g) were weighed and 10% polyvinyl polypyrrolidone was added. They were then separately ground in liquid nitrogen. The supernatants were transferred to other tubes, and five-fold 10% chilled tricarboxylic acid (TCA) acetone was added and kept at −20°C for 2 h. The precipitate was washed with chilled acetone for 30 min, and the supernatants were discarded after a 20,000 ×g centrifugation at 4°C for 20 min. This step was repeated three times. The pellets were air dried, and then dissolved in 500 μl 0.5 M triethylammonium bicarbonate (TEAB) buffer and sonicated at 200 Watts for 15 min. The samples were centrifuged at 30,000 ×g at 4°C for 20 min, and the supernatants were obtained. The disulfide bonds were reduced using 10 mM DTT at 56°C for 1 h. The cysteine was blocked with 55 mM iodoacetamide in a dark room for 45 min, then 5× chilled acetone was added and the samples were kept at −20°C overnight. The pellets were air dried, dissolved in 400 μl 0.5M TEAB and sonicated at 200 Watts for 15 min. The samples were centrifuged at 30,000 ×g at 4°C for 15 min. The supernatants were then collected. The protein concentration for each sample was measured using the 2-D Quant Kit according to the manufacturer’s protocol (GE Healthcare, Little Chalfont, UK) and detected by SDS-PAGE.

Protein digestion was performed according to the methods reported by Unwin et al. [46]. The samples were mixed with 10 mM DTT and incubated for 1 h at room temperature to reduce cysteine disulphide bonds. Then, 50 mM iodoacetamide was added and incubated for 1 h at room temperature in the dark. Subsequently, the samples were diluted by TEAB to reduce the urea concentration to ~1 M and 40 μg of trypsin was added to the samples. Then, the samples were incubated at 37°C overnight. After digestion, the samples were stored at −20°C or immediately labeled for iTRAQ.

### iTRAQ labeling

In total, 6 samples were included based on the characteristics of grain development for inbred N04. N1, N2 and N3 were from the pericarp and N4, N5 and N6 were from the endosperm at 10 DAP, 20 DAP and 33 DAP, respectively. The samples were divided into two groups based on the tissue. The pericarp group (Group 1) included N1–3 and the endosperm group (Group 2) included N4–6. For comparisons, N1 was also included in Group 2 and used as the control with reagent 113.

Proteins were labeled with the 8-plex iTRAQ reagents (Applied Biosystems) according to the manufacturer’s recommendations. The samples at 10 DAP, 20 DAP and 33 DAP were labeled with reagents 114, 115 and 116, respectively. The reactions were performed at room temperature for 1 h. All samples with equal fractions were collected and lyophilized using a SpeedVac, and then stored at −80°C.

### Two-dimensional Gel Electrophoresis

The isoelectric focusing (IEF) of the proteins from N04 endosperm at the three developmental stages was analyzed with three technical replicates. For each sample, equal amounts of total protein extract (1 mg) were assayed using 24 cm immobilized pH gradient (IPG) dry strips (Immobiline Dry-Strips, Bio-Rad, Hercules, CA, USA) with a linear pH gradient from 3 to 10. An active rehydration was performed overnight at 50 V. IEF was performed with an Ettan III system (GE Healthcare, USA) by increasing step by step from 50 to 10,000 V. Equilibration of the strips was performed immediately for 15 min with a buffer containing 0.375 M Tris-HCl (pH 8.8), 6 M urea, 20% glycerol, 4% SDS and 2% DTT, and followed for 15 min with a buffer containing 0.375 M Tris-HCl (pH 8.8), 6 M urea, 20% glycerol, 4% SDS and 2.5% iodoacetamide. Proteins from the IPG gel strips were separated using 12.5% SDS-PAGE gels. The gels were silver stained and scanned using a laser scanner (AlphaImager HP, Alpha Innotech, Santa Clara, CA, USA). Digital images of the gels were compared using Quantity One or PDQUEST software (Bio-Rad). The differentially expressed proteins (over two fold quantitative variations) were selected for further analyses. Differential spots in stained 2-dimensional gels were manually excised for protein digestion and LC-MS/MS analysis.

### LC-MS/MS analysis

The labeled samples were dissolved in 100 μl of strong cation exchange (SCX) chromatography buffer A, which contained 10 mmol/L KH_2_PO_4_ and 25% acetonitrile, pH 2.6. Each sample was separated into 20 gradients by the SCX column according to the spike and time, and separated using a reversed-phase column. The LC-MS/MS analysis was performed on a LTQ-Orbitrap-Velos system after desalinization. The MS spectra were received.

### Database search and protein identification

Tandem MS spectra raw data were used to performed ion peak detection, and peak listings were determined using the software Proteomics Tools [48]. Then, the raw data were transformed into the mgf format as the initial files. The software Mascot 2.3.02 (Matrix Science, http://www.matrixscience.com) was used to identify and quantify proteins. Searches were performed against the maize protein database released in January 2012 (http://www.plantgdb.org/ZmGDB/). The following search parameters were used: peptide mass tolerance ± 10 ppm, fragment mass tolerance ± 0.05 Da and maximum missed cleavages = 1. Trypsin was used as the enzyme with two missed cleavages. Fixed modifications were carbamidomethyl (C), iTRAQ8plex (N-term) and iTRAQ8plex (K). Variable modifications were Gln- > pyro-Glu (N-term Q), oxidation (M) and iTRAQ8plex (Y). Mass values were set as monoisotopic, instrument type as default and protein mass as unrestricted. The filter parameters for proteins were set as false discovery rate (FDR) ≤ 0.05, and for peptides, FDR ≤ 0.05. For relative protein quantitative analyses, the mass-to-charge ratio (m/z) 113 was performed as the control sample according to the peak area integral of mass-to-charge ratio (m/z) 113, 114, 115, and 116 reporter ions. The significant threshold values of ≥ 1.5 or ≤ 0.67 were regarded as differential protein expression.

### Function annotation and hierarchical cluster analysis of identified proteins

The functions of identified proteins were analyzed by matching to NCBInr (http://www.ncbi.nlm.nih.gov/) and/or Swiss-Prot/UniProt (http://www.uniprot.org/) databases, and further analyzed using the Gene Ontology (GO, http://www.geneontology.org) and the Cluster of Orthologous Groups (COGs, http://www.ncbi.nlm.nih.gov/COG/) of proteins databases. The metabolic pathways and signal transduction pathways of the identified proteins were analyzed according to the KEGG public database [57]. Hierarchical clusters of protein expression between the samples were performed by Cluster 3.0 software (http://bonsai.hgc.jp/~mdehoon/software/cluster/). The clustering results were viewed by TreeView software (http://bonsai.hgc.jp/~mdehoon/software/cluster/).

### Integrated network analysis on proteome data from both tissues of inbred N04

Because the expression intensities of proteins varied over time, protein candidates were filtered using expression intensity with the criterion of a fold change (absolute value) of no less than 5. Each protein was assigned a gene model from the MaizeGDB website. To annotate these proteins, a BLAST analysis was performed using the gene model sequences as query against the well-annotated maize orthology data of the KEGG database. Based on KEGG orthology, we linked the proteome data to KEGG pathways describing molecular interactions and reaction networks. Given a group of KEGG orthology entries, we used KEGG API (http://www.kegg.jp/kegg/rest/keggapi.html) to retrieve their related pathways with a URL format of http://rest.kegg.jp/link/ pathway/K01778 +K02725.

### Isolation of total RNA and mRNA

The samples were ground into powder and total RNA was isolated from the frozen samples using the hot phenol extraction method [58]. Reverse transcription was accomplished using the PrimeScript TM RT reagent Kit (Takara) to turn RNA to its complementary DNA (cDNA).

### Gene cloning and quantitative RT-PCR analysis

Four iTRAQ proteins for gene cloning were chosen based on their functions and differential expression levels in different tissue and stage samples, which were GRMZM2G060702 (Actin-depolymerizing factor, 3.6 for ratio N2/N3), GRMZM2G147687 (Exoglucanase1 precursor, 3.0 for ratio N3/N2 and 2.6 for ratio N3/N6), GRMZM2G111143 (Glucan endo-1,3-beta-glucosidase precursor, 3.1 for ratio N3/N2) and GRMZM2G102499 (General regulatory factor, 3.2 for ratio N6/N5). Each protein was used as a query against the National Center for Biotechnology Information (NCBI) EST database in a BLAST search. By assembling the overlapping ESTs into contigs, the complete open reading frame (ORF) was obtained. The assembled result was confirmed using the online tool Softberry to acquire annotations for the whole genome. Specific primers were designed using Primer 5.0 software to amplify the complete ORF. PCR was carried out using 1 μl of the obtained cDNA, 2.5 μl PCR buffer, 2.5 μl dNTPs mixture (2.5 mM each), 1 μl of each primer (10 μM), 0.125 μl Takara La Taq, and sterilized double-steamed water was added to a final volume of 25 μl. The PCR conditions were 1 min at 94°C then 30 cycles of 40 s at 94°C, 40 s at 56°C and 2 min at 72°C, and a final extension of 7 min at 72°C. PCR products were separated on 1% agarose gels and the single specific PCR product band was cloned into the pGEM-T easy vector (Promega) for sequencing.

Real-time PCR (RT-PCR) was performed using a Bio-Rad real-time detection system. The reaction liquid consisted of 12.5 μl 2× SYBR Premix Ex Taq II, 1 μl PCR forward primer (10 μM), 1 μl PCR reverse primer (10 μM), 2 μl of a 1/5 dilution of the cDNA as the template, and 8.5 μl sterilized distilled water. The total volume was 25 μl. The amplification procedure was as follows: 95°C for 3 min, then 40 cycles each of denaturation at 95°C for 10 s, annealing at 58°C for 20 s, and extension at 72°C for 30 s. The β-actin gene was used as the control, and each sample was repeated three times. Template cDNAs were obtained from the reverse transcription of the RNA extracted from collected samples. The area under the curve for the PCR product of each nucleotide was compared to that of its respective internal standard (Ct) to determine gene expression values [58]. The Ct values were the means of the three repeats. If the differences among them exceed 0.5 Ct, then they were discarded [59]. Relative expression volume (REV) was computed as 2^(−ΔΔCt)^ according to the method by Ganeshan et al. [60], in which ΔΔCt = ΔCt–ΔCt (calibrator), ΔCt = Ct (sample) − Ct (reference gene).

Primers used for RT-PCR analysis included as follows:

GRMZM2G060702, 5'-CCCCGAGAATGACTGCCGATAC-3' and 5'-TTCACCTTGGCGGAGGATGG-3';

GRMZM2G147687, 5'- GCCGGCCGTCGACACATGTT-3' and 5'- TGGGCTCCGGTAGTGTCGCCT-3';

GRMZM2G111143, 5'-AGCGGAACTTCGGCATCTT-3' and 5'-CACGCACCACTTGCCTCC-3';

GRMZM2G102499, 5'-TGCTGTCTTGATTTCGGGTCG-3' and 5'-AGCGTCGCACCCAAGAAAC-3'.

### Western blot analysis

The same four proteins analyzed by RT-PCR were chosen for Western blot analysis. Total proteins for all samples as in iTRAQ were extracted by a method combining the usage of Borax/PVPP/Phe (BPP) as described by Wang et al. [61]. 5 μg of the actin and the total sample proteins were separated by 12% (w/v) sodium dodecyl sulphate polyacrylamide gel electrophoresis (SDS-PAGE), and transferred onto PVDF microporous membranes [62]. Blocking for 2.5 h in TBST buffer(20 mM TrisHCl, pH 7.6, 150 mM NaCl, and 0.05% Tween 20) with 5% nonfat dry milk at room temperature, membranes were incubated with the special antibodies of the four proteins at 1:1,000 dilution for overnight at 4°C. Custom rabbit polyclonal antisera for 4 select proteins were produced by Abmart Inc. (Shanghai, China). Following three times washing with TBST, membranes were incubated with secondary antibodies (Goat anti-Rabbit IgG-HRP, Abmart) at 1:5,000 dilution for 1.5 h at room temperature away from light. After three washings with TBST, signals were detected using an ECL Western Blotting Kit (Amersham) following the manufacturer’s instructions. The ratio of the validation proteins were compared to actin (#M20009, Abmart) and were densitometric measured by Image J software (NIH, Bethsda, MD).

## Results

### The dynamic developmental process of grain and its two components

**Fig 1:**
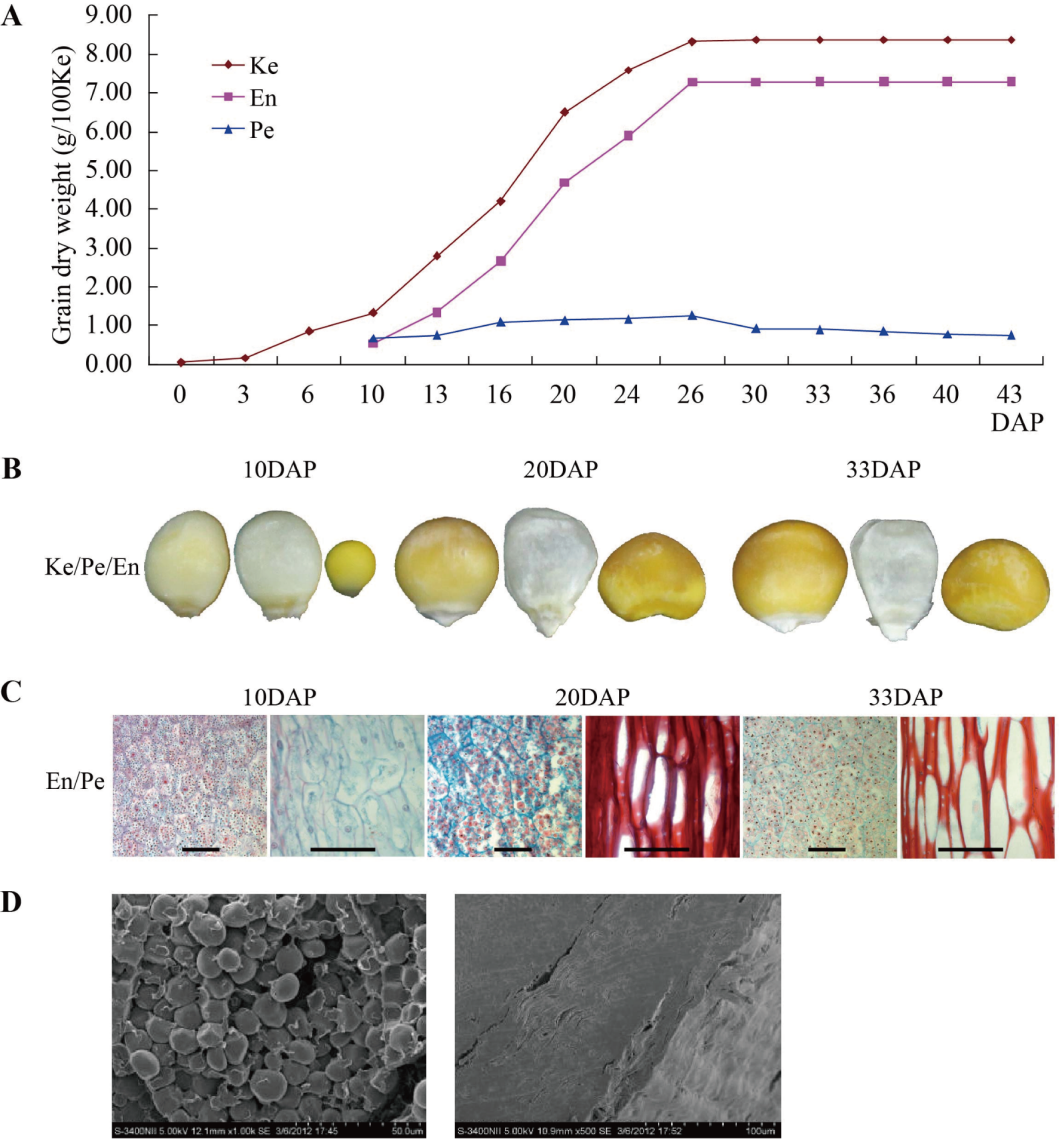
The developmental pattern and the morphological and cytological characteristics of kernels from maize inbred line N04. A. The developmental patterns of grain and its two component parts, the endosperm and pericarp. B. The exact appearance for complete kernel (Ke), endosperm (En), and pericarp (Pe) at 10, 20 and 33 DAP for N04. C. Microscopic section of pericarp (right) and endosperm (left) during kernel development. D. The characteristics of pericarp (the right) and starch granules in the endosperm (the left) for N04 middle maturity kernels by scanning electron microscopy. (B) bar = 8 cm, (C, D) for endosperm, bar = 50 μm and for pericarp, bar = 100 μm, respectively.

After pollination, the weights for the whole grain from 3 to 43 DAP, and for the two component parts (endosperm and pericarp) from 10 to 43 DAP for inbred N04, were quantified respectively. The whole grain weight increased slowly before 10 DAP, followed a rapid increase during 10–26 DAP, and then increased slowly again (Fig 1A and 1B). The same pattern was observed for the two components. At 33 DAP, both the whole grain and endosperm weights reached the highest point, which were 8.36 g/100 grains and 7.29 g/100 grains, respectively.

Microstructures of the endosperm and pericarp at 10, 20 and 33 DAP showed that the cell sizes of the endosperm and pericarp changed in a similar manner at all comparable developmental stages (Fig 1C). Endosperms at 10 DAP were in the cell division stage, and then, the endosperm cells accumulated substances rapidly. At 20 DAP, endosperm cells were filled with starch grains.

Using light microscopy and SEM highly different characteristics were observed for starch granules in endosperm and pericarp at maturity (Fig 1C and 1D). The pericarp texture was rough. The starch granules in the endosperm were small polygonal, densely packed and filled with protein bodies. Simultaneously considering the development patterns and the structures of the whole grain, the endosperm and pericarp at 10, 20 and 33 DAP were chosen for further analyzing the differentially expressed proteins during grain development.

### Quantitative protein identification of endosperm and pericarp using iTRAQ

**Fig 2:**
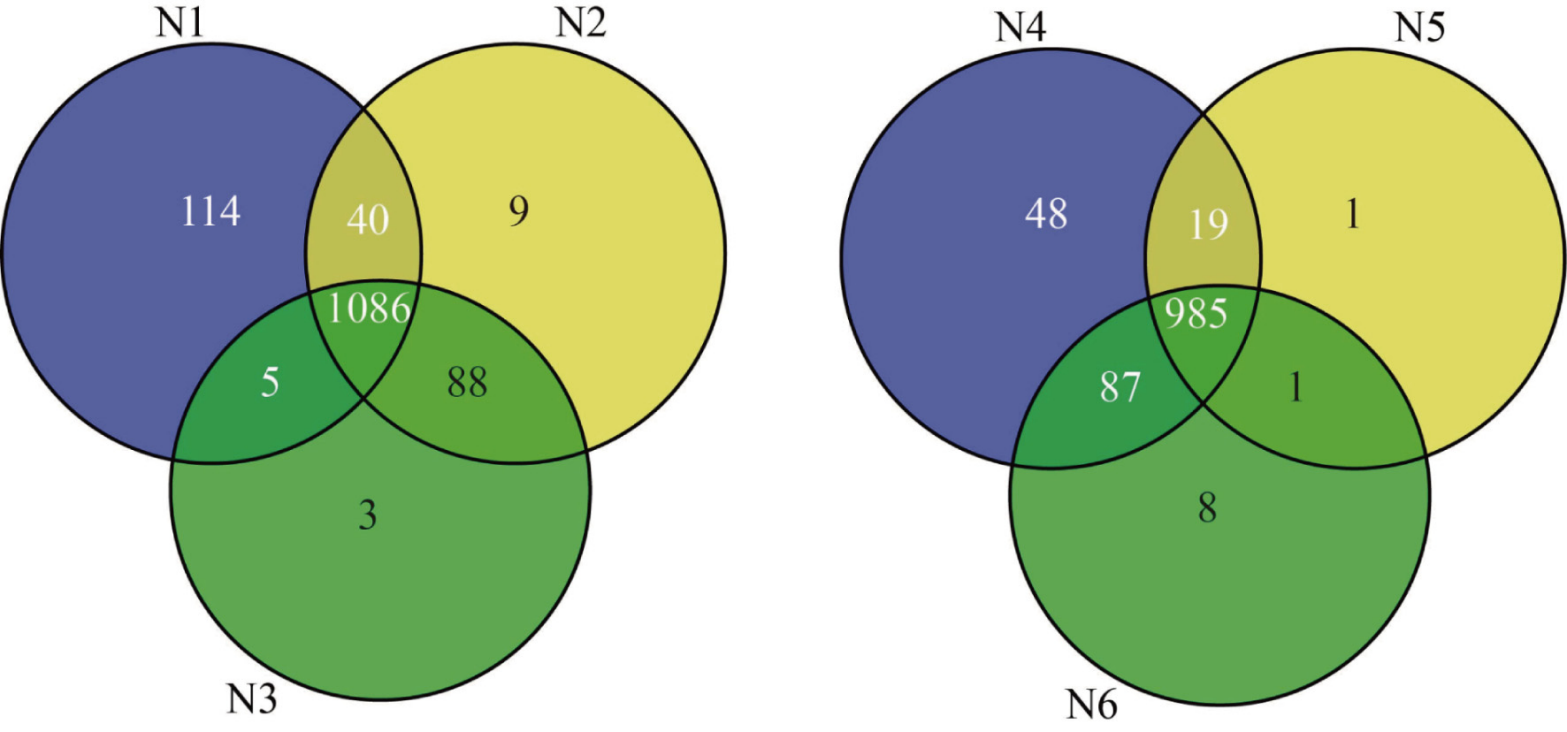
Quantities of differentially expressed proteins according to the developmental stages of the two tissues for the maize inbred line N04. Venn diagrams were used to identify proteins in the pericarp(left) and endosperm(right), respectively.

A total of 6,876 proteins were identified in all samples, 1,345 from pericarp and 1,149 from endosperm, among which 1,396 were nonredundant proteins (Table 1, Fig 2). The peptides and the quantitation information of identified proteins were listed in S1 File. There were 227 uniquely identified proteins from pericarp and 46 from endosperm (Table A in S2 File). For the pericarp, a total of 3,650 proteins were obtained, 1,245, 1,223, and 1,182 proteins were recognized at the three stages, 10 DAP, 20 DAP and 33 DAP, respectively, of which 1,086 proteins were commonly found at all developmental stages, and 114, 9 and 3 proteins were stage specific, respectively. For the endosperm, a total of 3,226 proteins were obtained, 1,139, 1,006 and 1,081 proteins were recognized at 10 DAP, 20 DAP and 33 DAP, respectively, of which 985 proteins were commonly found at all developmental stages, and 48, 1 and 8 proteins were stage specific, respectively.

**Table 1:** The information of proteins identified using iTRAQ.

Inbred	Tissue	10DAP	20DAP	33DAP	Total	Nonredundance
N04	Pericarp	1245	1223	1182	3650	1345
	Endosperm	1139	1006	1081	3226	1149
Total		2384	2229	2263	6876	—
Nonredundance		1367	1360	1370	—	1396

### Functional annotation of identified proteins using iTRAQ

**Fig 3:**
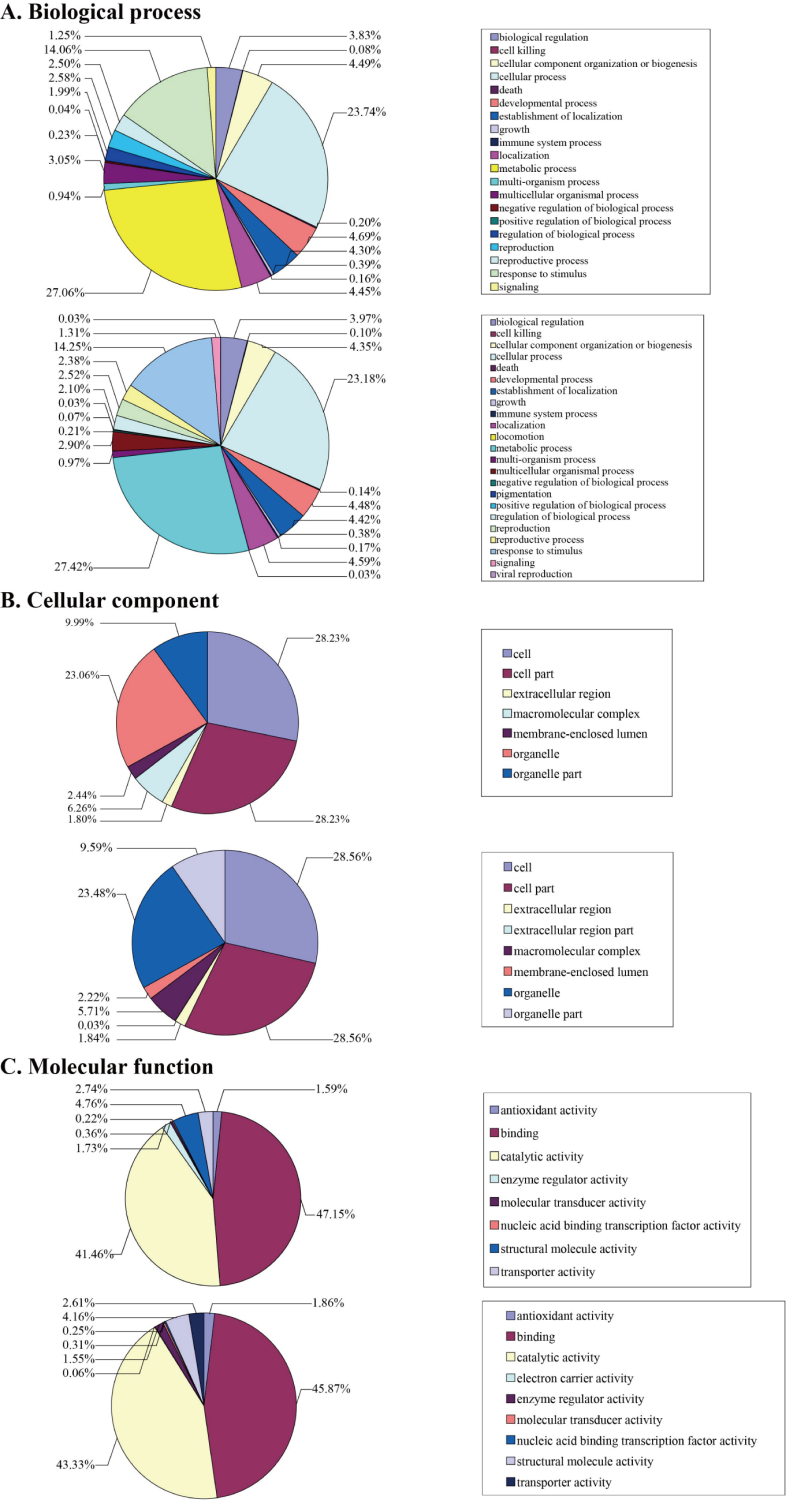
Functional classification of the identified proteins at all developmental stages for both endosperm (above) and pericarp (below) of maize inbred line N04 according to GO annotations in biological process (A), molecular function (B) and cellular component (C).

Biological process of the endosperm proteins could be classified into 20 categories according to gene ontology (GO) (Table B in S2 File, Fig 3). The largest functional category was related to metabolic process (27.06%), and the second category was cellar process (23.74%). Proteins involved in other biological processes were related to stimulus response (14.06%), developmental process (4.69%), localization (4.45%), cellular component organization or biogenesis (4.49%), establishment of localization (4.30%), biological regulation (3.83%) and multicellular organismal process (3.05%). For the molecular function annotation, eight GO terms were enriched, mostly including binding (47.15%) and catalytic activity (41.46%). For the cellular component annotation, seven GO terms were enriched, which mostly included cell (28.23%), cell part (28.23%) and organelle (23.06%).

For pericarp proteins, 23 GO terms were enriched for biological processes. The largest seven functional categories were also associated with metabolic process (27.42%), cellar process (23.18%), stimulus response (14.25%), localization (4.59%), developmental process (4.48%), cellular component organization or biogenesis (4.35%) and establishment of localization (4.42%), as was seen for the endosperm. For molecular function, binding (45.87%) and catalytic actively (43.33%) were the most abundant. For cellular component, cell (28.56%), cell part (28.56%) and organelle (23.48%) made up the majority.

To more accurately annotate the functions of proteins in the pericarp and endosperm, all proteins were further aligned to the COG database for functional prediction and classification. For the endosperm proteins, 983 proteins were successfully functionally annotated, and they were involved in 22 metabolic processes (Fig A in S2 File). The majority of the abundant proteins for endosperm development were related to posttranslational modification, protein turnover or chaperones (17.60%), translation, general function prediction only (14.04%), ribosomal structure and biogenesis (12.21%), followed by energy production and conversion (11.19%), carbohydrate transport and metabolism (10.48%), amino acid transport and metabolism (9.46%), and lipid transport and metabolism (3.46%). All key enzymes involved in the TCA cycle and glycolysis were identified, which played important roles in kernel development in plant.

In total, 1107 pericarp proteins were successfully functionally annotated and were involved in 22 metabolic processes. The majority of the abundant proteins for pericarp development were related to posttranslational modifications, protein turnover or chaperones (15.72%), general function prediction only (14.00%), energy production and conversion (11.74%), translation, ribosomal structure and biogenesis (10.84%), carbohydrate transport and metabolism (10.57%), amino acid transport and metabolism (9.76%), followed by lipid transport and metabolism (4.52%), and cell wall/membrane/envelope biogenesis (2.98%). Cell cycle control, cell division and chromosome partitioning (0.63%), RNA processing and modification (0.18%) and nuclear structure (0.09%) were the smallest groups in pericarp and endosperm.

### The differentially expressed proteins across different developmental stages and tissues

Distinct differences in protein expression profiles were observed across different developmental stages and tissues, especially between the two tissues (Fig 3, Table C in S2 File). For the differentially expressed proteins among developmental stages, the number of pericarp proteins with the ratios of increased/decreased expression levels were 168/226 and 352/378 for N2/N1 and N3/N2, respectively. For endosperm proteins, those ratios were 285/243 and 251/456 for N5/N4 and N6/N5, respectively. Over 300 proteins showed different expression levels between the two tissues at each developmental stage. The number of differentially expressed proteins between the two tissues having the ratios of increased/decreased expression levels were 442/486, 426/364 and 371/377 for N4/N1, N5/N2 and N6/N3, respectively.

**Fig 4:**
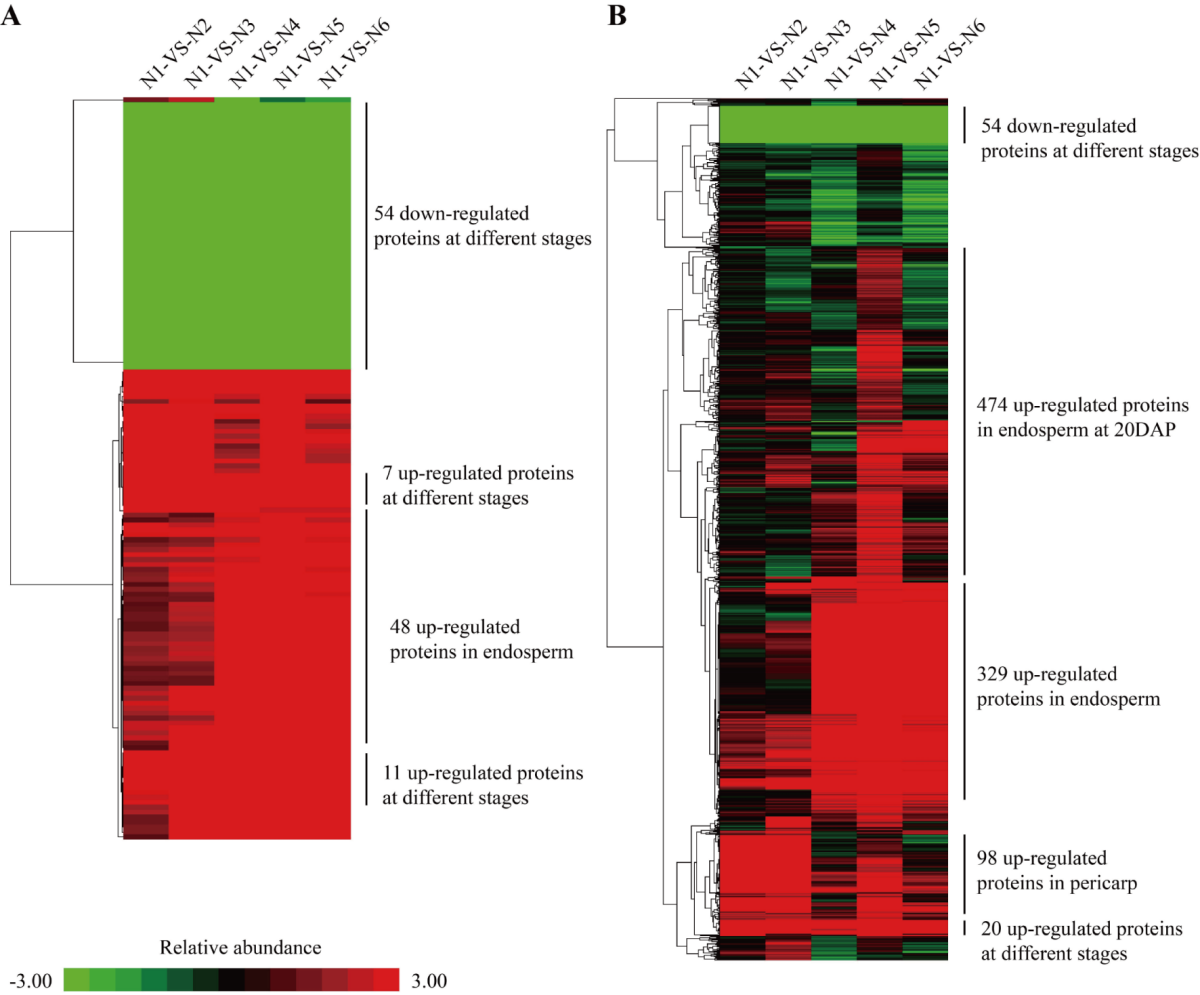
Hierarchical clustering analysis for the patterns of differentially expressed proteins in maize kernel development. A. intersection; B. Union.

The main functions of the differentially expressed proteins were almost the same across all comparisons, relating to posttranslational modification, protein turnover and chaperones, general prediction function, carbohydrate transport and metabolism, amino acid transport and metabolism, energy production and conversion, and translation, and ribosomal structure and biogenesis. The differentially expressed proteins among different developmental stages showed that the proteins were mainly divided into two types, endosperm and pericarp, by intersection analysis (Fig 4A). For the two tissues, their protein expression profiles were obviously different. Most of the proteins were up-regulated at 10 DAP in pericarp, otherwise, a majority of proteins were up-regulated at 20 DAP in endosperm. Totally, 54 proteins with down-regulation and 18 proteins with up-regulation in both tissues at three developmental stages were observed. The functions for the 54 proteins with down-regulation were mainly involved in carbohydrate transport and metabolism, energy production and conversion and posttranslational modification, protein turnover, chaperones pathways, and the functions for the 11 proteins with up-regulation were posttranslational modification, protein turnover, chaperones, translation, ribosomal structure and biogenesis, and general function prediction only, and the functions for the seven proteins with up-regulation were cell wall/membrane/envelope biogenesis and mino acid transport and metabolism. In addition, 48 proteins were up-regulated only in endosperm, which were involved in cell wall/membrane/envelope biogenesis, amino acid transport and metabolism, translation, ribosomal structure, biogenesis and general function prediction.

Furthermore, similar result was obtained using a union analysis of differential expression proteins (Fig 4B). Fifty-four proteins were down-regulated, and 20 proteins were up-regulated in both tissues, which were involved in energy production and conversion, amino acid transport and metabolism, translation, ribosomal structure and biogenesis, posttranslational modification, protein turnover and chaperones. While, 474 proteins were up-regulated at 20 DAP in endosperm, for which their functions were energy production and conversion, amino acid transport and metabolism, posttranslational modification, protein turnover, chaperones and translation, ribosomal structure and biogenesis. In addition, 329 proteins up-regulated in endosperm with functions in energy production and conversion, amino acid transport and metabolism, carbohydrate transport and metabolism, lipid transport and metabolism, translation, ribosomal structure and biogenesis, posttranslational modification, protein turnover, and chaperones, and 98 proteins up-regulated in pericarp with functions in posttranslational modification, protein turnover, chaperones, amino acid transport and metabolism, translation, ribosomal structure and biogenesis, chromatin structure and dynamics, secondary metabolites biosynthesis, transport and catabolism, were observed at three developmental stages.

### Protein network integration on obtained proteome data

**Fig 5:**
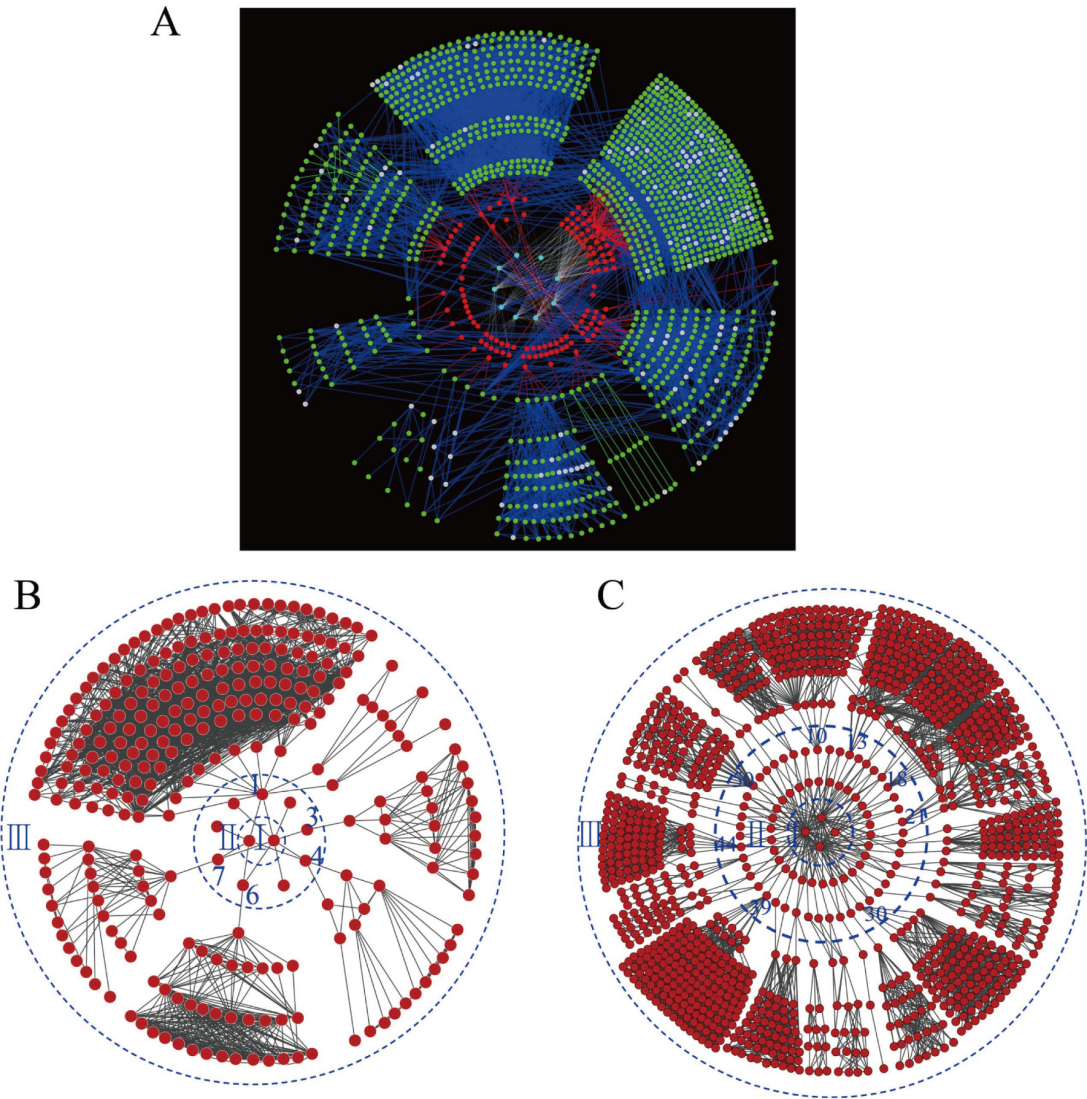
A complex network of 125 proteins (A), proteins expressed in the pericarp only (B) and endosperm only (C) from two maize inbred lines at respective time points. In network A, the red nodes represent proteins from our experiments, while green nodes represent annotated orthologs or enzymes from the KEGG database. Gray nodes represent metabolites or compounds, green edges represent protein interactions, and red edges represent relations between our investigated proteins and entries from the KEGG database. In networks B and C, the red nodes in circles I, II and III indicate time points, differentially expressed proteins identified in this study, and interacting network proteins from the KEGG database.

To obtain the specific proteins associated with kernel development, the biological networks of all identified proteins (Fig 5A), only the pericarp proteins (Fig 5B) and only the endosperm proteins (Fig 5C) were constructed based on protein interactions. Out of the 1,396 investigated proteins, 1,034 proteins were linked to KEGG orthology by constraining BLAST hits with an e-value ≤ 1e-05. There were 125 proteins significantly expressed in 50 groups of comparisons between any two time-points with a criterion of fold change no less than five (Table 2). The gene model sequences of these proteins were run against KEGG orthology using BLAST to relate proteins to the functional pathway maps from the KEGG database. Fig 5A shows a complex network of 125 proteins differentially expressed at the respective time points. Those differentially expressed proteins were involved in 54 KEGG pathways. All the network clusters were related to the same biological processes. The top 10 pathways with the largest numbers of proteins were shown in Table 3, revealing their importance during the development of endosperm and pericarp in maize.

**Table 2:** Numbers of differentially expressed proteins at each time point.

Time points	Proteins	Time points	Proteins
N6	56	N4	39
N5/N3	54	N4/N2	36
N5/N2	49	N6/N3	34
N6/N2	45	N3	8
N4/N3	43	N3/N2	5
N5	41	N6/N4	4

Note: At all single time points, the fold change of expression intensity were calculated by subtracting expression values at N1 from those time points. For example, at N6, there are 56 proteins with expression values at least 5 fold different from those at N1. At double time points, fold change is calculated as the difference between the two time points.

**Table 3:** Top 10 pathways involving the most proteins.

Pathway ID	Description	Proteins
path:ko00290	Valine	16
path:ko00670	One carbon pool by folate	15
path:ko00500	Starch and sucrose metabolism	8
path:ko00410	beta-Alanine metabolism	7
path:ko00260	Glycine	7
path:ko00010	Glycolysis / Gluconeogenesis	7
path:ko00562	Inositol phosphate metabolism	7
path:ko04910	Insulin signaling pathway	7
path:ko00280	leucine and isoleucine degradation	7
path:ko03015	mRNA surveillance pathway	7

Of the two tissues, more differentially expressed proteins were integrated in the endosperm network (77) than in the pericarp network (9) (Fig 5B and 5C; Table D in S2 File). In the pericarp protein network, 15 proteins with first-degree interactions and 255 proteins with second-degree interactions were found. For the endosperm network, 108 proteins with first-degree interactions and 1000 proteins with second-degree interactions were found. However, the functions of 4 proteins in the pericarp network and 44 proteins in the endosperm network were unknown.

### Confirmation of proteins from iTRAQ by 2DE, relevant gene cloning and expression analysis, and western blot

To verify the proteins identified by iTRAQ, the endosperm proteins at 10, 20 and 33 DAP for inbred N04 were separated by 2DE technology (Fig B in S2 File). In total, 47 protein spots with obvious differences were identified at a pH range from 3 to 10. They were digested with trypsin and analyzed by LC-MS/MS. By matching to the NCBInr database, 36 spots were processed successfully and their corresponding proteins were identified (Table E in S2 File). The majority of the proteins were related to posttranslational modification, carbohydrate transport and metabolism, amino acid transport and metabolism, and energy production and conversion. Compared with the quantitative endosperm proteins identified by iTRAQ, 16 proteins were commonly found, accounting for 44.44%.

To compare the expression patterns of iTRAQ proteins at the RNA level identified by iTRAQ were selected based on their expression differences among different samples and predicted functions (Fig C in S2 File). The relevant genes were cloned and RT-PCR was performed. The results showed that the concordance between mRNA level and the protein abundance varied across different proteins, tissues and developmental stages (Fig C in S2 File). No complete concordance was found for any proteins.

For the western blot, we characterized the four proteins from iTRAQ by using actin as control (Fig C in S2 File). Comparison of the relative abundances of the four proteins between iTRAQ and western blot showed that all the same tendencies were observed. Although the expression level of protein GRMZM2G147687 was different in pericarp, but it was not significantly different between both methods.

## Discussion

Maize kernel development was affected by multiple genes with dynamic expression levels through complex physiological and biological processes [3, 4, 6]. Proteome maps have become powerful tools for physiological studies of maize kernel development. In this research, the dynamic proteome of two kernel tissues, endosperm and pericarp, were analyzed by iTRAQ labeling in popcorn. The result by western blot for four proteins showed the same expression tendency as in iTRAQ. The protein regulation characteristics during the development of the endosperm and pericarp were uncovered.

### Endosperm proteins involved in cell division and differentiation

Cell division, cell elongation and cell differentiation play critical roles in the kernel developmental process [20, 63]. Hormones, tubulins, actins, dehydroascorbate reductase, ras-related proteins and heat shock proteins are all important in cell cycle control and cell proliferation during plant development [20, 64–65]. In this study, 102 proteins directly involved in regulating cell division, cell elongation and cell differentiation were identified at 10 DAP (Table F in S2 File). In addition, a polypeptide, cellular apoptosis susceptibility protein/chromosome-segregation protein (GRMZM5G881950), which is involved in chromosome segregation, controlling cell proliferation and intracellular protein transport, was identified in the endosperm. This protein showed much higher expression level at 10 DAP than at 20 DAP in endosperm. Those proteins involved in cell division and differentiation showing obvious high expression levels at 10 DAP endosperm compared with that at 20 DAP endosperm, together with their specific proteins, might play important roles in resulting in the contrasting cell number and cell size of kernels.

### Proteins involved in storage product accumulation during endosperm development

The dry-matter of the maize endosperm mainly accumulated during 12–35 DAP, which was greatly different to the grain-filling periods of various inbreds/hybrids [4, 15]. Generally, starch, which accounts for ~75% of the endosperm dry-matter, could be detected at 9–12 DAP and accumulated rapidly in the late stages [4, 5]. In this study, the fast grain filling period was 10–26 DAP for popcorn inbred N04. Forty-five proteins directly involved in starch biosynthesis and metabolism were identified in the endosperm (Table G in S2 File). The protein expression patterns in starch biosynthesis showed that 19 proteins having increased expression levels between 20 and 33 DAP in endosperm, reflecting the important role of this stage in determining kernel weight.

In fact, starch biosynthesis and accumulation is a very complex biochemical process. Several complex and inter-related metabolic processes are involved in maize kernel development to maintain a normal metabolism, including amino acid synthesis, carbon metabolism, storage starch and protein metabolism [3,13,15–17,29]. In this study, large quantities of proteins for amino acid synthesis (79), carbon metabolism (84), and energy production and conversion (79) were identified in the endosperm, reflecting their important roles in endosperm development.

The TCA cycle is not only a fundamental metabolic pathway for energy production, it also provides the substrates for amino acid synthesis or interconversion [66]. Certain amino acids can enter the TCA cycle [64–65]. Acetyl Coenzyme A (acetyl-CoA) is the important intermediate metabolite of carbohydrates, lipids and amino acids that plays a key role in the TCA cycle [67–68]. In this study, 46 acetyl-CoA related enzymes were recognized, including acetyl-CoA acetyltransferase (2), acetyl-CoA carboxylase (2), acyl-CoA dehydrogenase (2), acyl-CoA synthetase (2) and acyl-CoA-binding protein (1), citrate synthase (3), aconitase (3), isocitrate dehydrogenase (6), oxoglutarate dehydrogenase complex (16), succinyl-CoA synthetase (2), succinate dehydrogenase (2) and malate dehydrogenase (5).

### Proteins related to the formation and structure of the kernel pericarp

The pericarp plays two important roles during grain development, one is protecting the embryo and endosperm, and the other is the limiting grain expansion through extensibility and thickness [22]. A series of complex physiological and biochemical processes also takes place during the pericarp development.

The pericarp texture for the popcorn inbred N04 was very compact. Previous research had shown that popcorn varieties accumulate more extensin in the pericarp, which results in its tough texture. Additionally, the increase in extensin content paralleled the increase in whole kernel dry weight during kernel development [27]. In this study, 4 leucine-rich repeat extensin-like proteins were identified in the pericarp tissue (Table H in S2 File), which could modulate cell morphogenesis by regulating cell wall formation and assembly. An increase in the expression of extensin-like proteins at late stages was beneficial for the formation of high extension. Those specific proteins and highly expressed proteins in the pericarp might simultaneously determine its special structure.

### Protein functions and interactions revealed through their network integration

Plant tissues undergo a series of dynamic physiological and biochemical processes to maintain its growth and development, which involve thousands of different proteins. Therefore, it is fairly difficult to reveal the possible relationships among these proteins. With the development of biological technologies, gene and protein expression profiles could be identified [30–31]. A metabolic network reconstruction could uncover the molecular mechanisms of a particular organism, which would be beneficial to unraveling the systems biology of metabolic pathways [32–33]. This would further contribute to the identification of key genes or proteins that play important roles in the biological and chemical processes during plant development [19].

In this research, the protein regulatory networks related to kernel development were constructed based on the KEGG orthology system (Fig 5A). The functions of the differentially expressed proteins and their relationships were revealed. Also, the regulatory networks uncovered the differences between tissues, and the dynamic changes in protein expression level during kernel development. Of the 9 proteins in the pericarp protein network (Fig 5B), five proteins had first-degree interaction proteins. Three proteins each only had one first-degree interaction protein, one protein had two first-degree interaction proteins, and one protein had ten first-degree interaction proteins. The function for protein No.4 (GRMZM2G005633_P02) was uncharacterized protein contain chitin-binding domain type 3, which had two first-degree interaction proteins and took part in starch and sucrose metabolism. The gene to code this protein was named as *ZmCHI-B*, which regulated pericarp development [69].

For the 77 proteins in the endosperm protein network (Fig 5C), 33 proteins had first-degree interaction proteins. Protein No. 50 (GRMZM2G132903), Proteins No. 39 (GRMZM2G339994), proteins No. 13 (GRMZM2G008714), proteins No. 18 (GRMZM2G097226) and proteins No. 44 (GRMZM2G001898), proteins No. 10 (GRMZM2G135283), proteins No. 21 (GRMZM2G154595), 30 (GRMZM2G090087) and 43 (GRMZM2G445905) had ten, eight, seven, six and five first-degree interaction proteins, respectively. Protein No. 50 (GRMZM2G132903), which had the same function as protein No.1 (GRMZM2G339994) in the pericarp protein network, had the most of first-degree interaction proteins (23 proteins). The function for this protein was 3-hydroxyacyl-CoA dehydrogenase, which took part in alpha-linolenic acid metabolism and fatty acid metabolism [70–71]. The function for GRMZM2G339994_P01 was SDR dehydrogenases with different specificities (related to short-chain alcohol dehydrogenases), which had 10 first-degree interaction proteins and took part in fatty acid biosynthesis and biotin metabolism [72]. Protein GRMZM2G090087_P02, with the function of delta 1-pyrroline-5-carboxylate dehydrogenase, had five first-degree interaction proteins and took part in alanine, aspartate, glutamate, arginine and proline metabolism [73–74]. In addition, 24 other proteins had three two and one first-degree interactions, respectively. Our future research would concentrate on the functional verification of these proteins and their interaction proteins.

### Non-concordance of gene expression at RNA and protein levels

Proteins represent the end-products of the functional translations of genes. However, high levels of non-concordance between RNA and protein expression levels have been observed in several previous studies [37–38, 31, 36]. In the endosperm of inbred B73, ~22% of the genes showed matching rank abundances between their mRNA and protein using MS and the NimbleGen microarray [31]. In the developmental maize leaf blade gradient, 13% of the genes detected by RNA-seq were identified at the protein level, whereas 99% of all detected proteins were identified at the mRNA level using label-free shotgun proteomics and transcript data based on RNA-seq [39]. In our present study, four proteins identified by iTRAQ were selected to reveal their expression tendency at the mRNA level. The results showed that no complete concordance was found for any proteins.

Several reasons might cause a high non-concordance between mRNA and protein levels, such as posttranscriptional regulation [37], posttranslational modification, and differential protein and mRNA degradation rates [38–39], codon bias, UTRs and length of mRNAs, and protein functions. Considering that the gene expression at protein level might reflect the real function of genes, temporal and spatial proteins should be extensively detected to thoroughly reveal the genes in influencing kernel development in maize.

## Supporting Information

S1 File Supplementary dataset. Sheet 1. The peptides of identified proteins using iTRAQ; Sheet 2. The quantitation of identified proteins in endosperm and pericarp using iTRAQ.
(XLS)

S2 File Supplementary Figs and Tables. **Table A.** Specific proteins identified only in endosperm and pericarp for inbred line N04. **Table B.** Protein functional-categories identified by Gene Ontology (GO) analysis for pericarp and endosperm of inbred line N04 at all developmental stages. **Table C.** Quantities of the differentially expressed proteins by contrast across different developmental stages and tissues. **Table D.** The functions of differentially expressed proteins integrated in the endosperm and pericarp networks. **Table E.** Differentially expressed proteins identified by 2-DE. **Table F.** The functions and expression differences of inbred line N04 for endosperm proteins directly involved in regulating division, elongation and differentiation at 3 developmental stages. **Table G.** The functions and expression differences of inbred line N04 for 45 endosperm proteins directly involved in starch biosynthesis and metabolism at 3 developmental stages. **Table H**. The functions and expression differences of inbred line N04 for proteins directly related with the formation and structure of kernel pericarp. **Fig A.** Protein functional categories annotated for pericarp (A) and endosperm (B) of inbred line N04 according to assignments of cluster of orthologous groups (COG). **Fig B.** 2-DE protein map of 20DAP endosperm for inbred line N04. **Fig C.** Real-time PCR and Western blot analysis of four proteins from iTRAQ in endosperm and pericarp at developmental stages for inbred line N04. (A) Relative abundance of the protein from iTRAQ, and all the groups were controled by pericarp at 10 DAP. (B) Real-time PCR for four proteins mRNA expression in IL N04 at 10 DAP, 20 DAP and 33 DAP. (C) Western blot analysis for the proteins after separation on SDS-PAGE, the proteins were controled by actin. VEPI, Validated the Expression of the Proteins by using iTRAQ. The results are presented as means+SEM pooled from three independent experiments in B and C.
(DOC)
