# Supplementary material for: Dynamic Proteomic Characteristics and Network Integration Revealing Key Proteins for Two Kernel Tissue Developments in Popcorn
Source: PLoS One. 2015 Nov 20;10(11):e0143181. doi: 10.1371/journal.pone.0143181 (PMC4654522; doi:10.1371/journal.pone.0143181)
Supplement: S2 File — Table A. Specific proteins identified only in endosperm and pericarp for inbred line N04. Table B. Protein functional-categories identified by Gene Ontology (GO) analysis for pericarp and endosperm of inbred line N04 at all developmental stages. Table C. Quantities of the differentially expressed proteins by contrast across different developmental stages and tissues. Table D. The functions of differentially expressed proteins integrated in the endosperm and pericarp networks. Table E. Differentially expressed proteins identified by 2-DE. Table F. The functions and expression differences of inbred line N04 for endosperm proteins directly involved in regulating division, elongation and differentiation at 3 developmental stages. Table G. The functions and expression differences of inbred line N04 for 45 endosperm proteins directly involved in starch biosynthesis and metabolism at 3 developmental stages. Table H. The functions and expression differences of inbred line N04 for proteins directly related with the formation and structure of kernel pericarp. Fig A. Protein functional categories annotated for pericarp (A) and endosperm (B) of inbred line N04 according to assignments of cluster of orthologous groups (COG). Fig B. 2-DE protein map of 20DAP endosperm for inbred line N04. Fig C. Real-time PCR and Western blot analysis of four proteins from iTRAQ in endosperm and pericarp at developmental stages for inbred line N04. (A) Relative abundance of the protein from iTRAQ, and all the groups were controled by pericarp at 10 DAP. (B) Real-time PCR for four proteins mRNA expression in IL N04 at 10 DAP, 20 DAP and 33 DAP. (C) Western blot analysis for the proteins after separation on SDS-PAGE, the proteins were controled by actin. VEPI, Validated the Expression of the Proteins by using iTRAQ. The results are presented as means+SEM pooled from three independent experiments in B and C. (DOC) [file pone.0143181.s002.doc]

**Supplemental Figures and Tables**


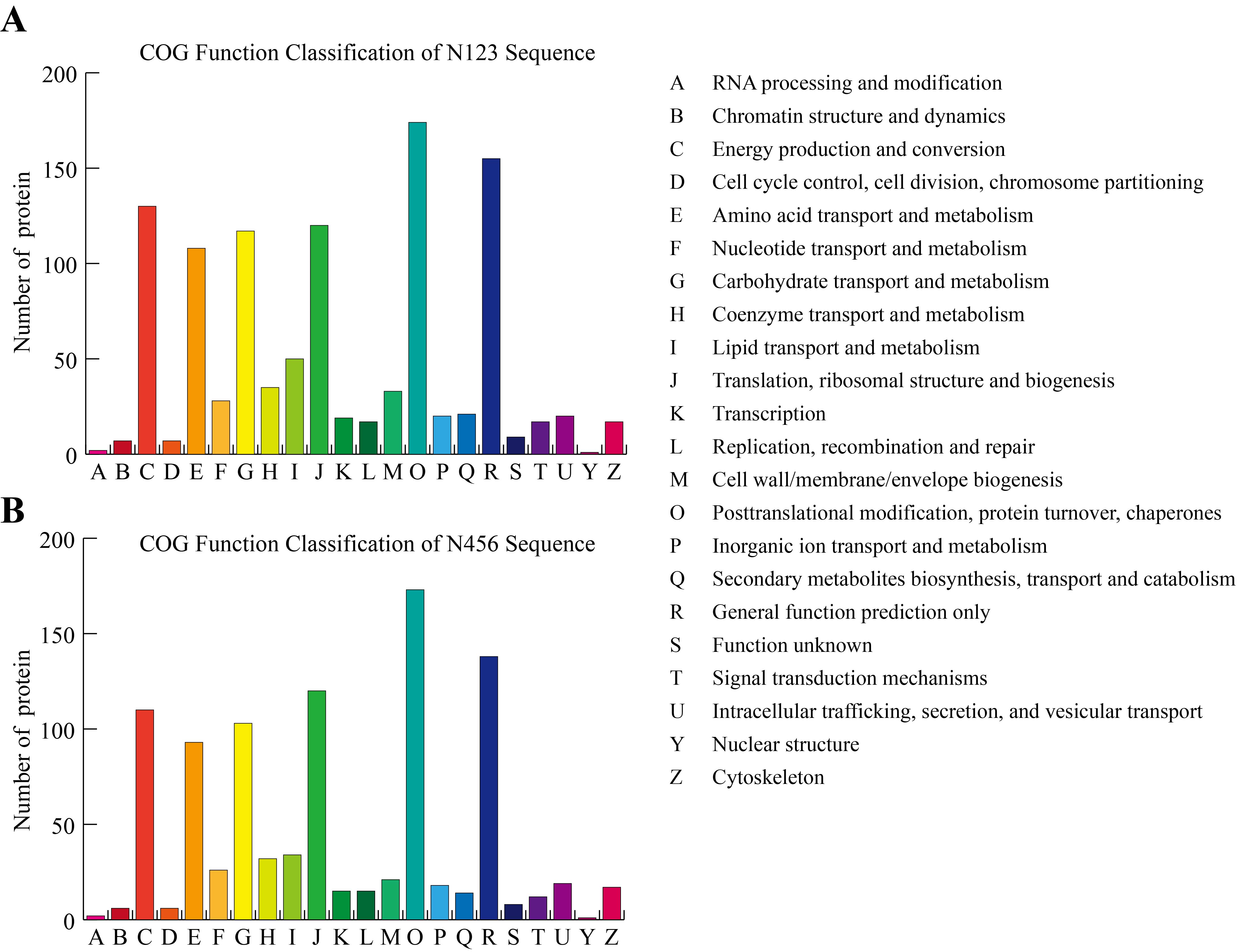


**Fig. A.** Protein functional categories annotated for pericarp (A) and endosperm (B) of inbred line N04 according to assignments of cluster of orthologous groups (COG).


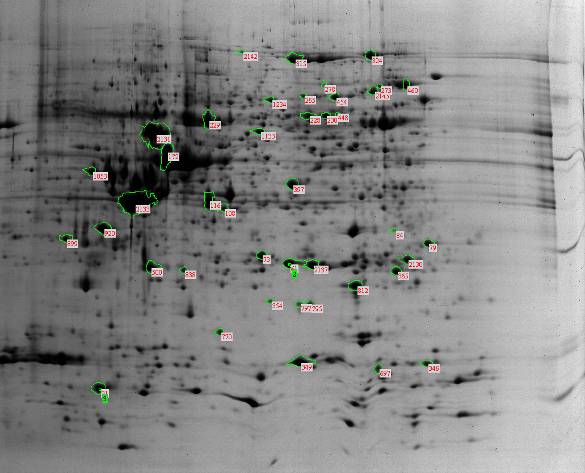


**Fig. B.**  2-DE protein map of 20DAP endosperm for inbred line N04.


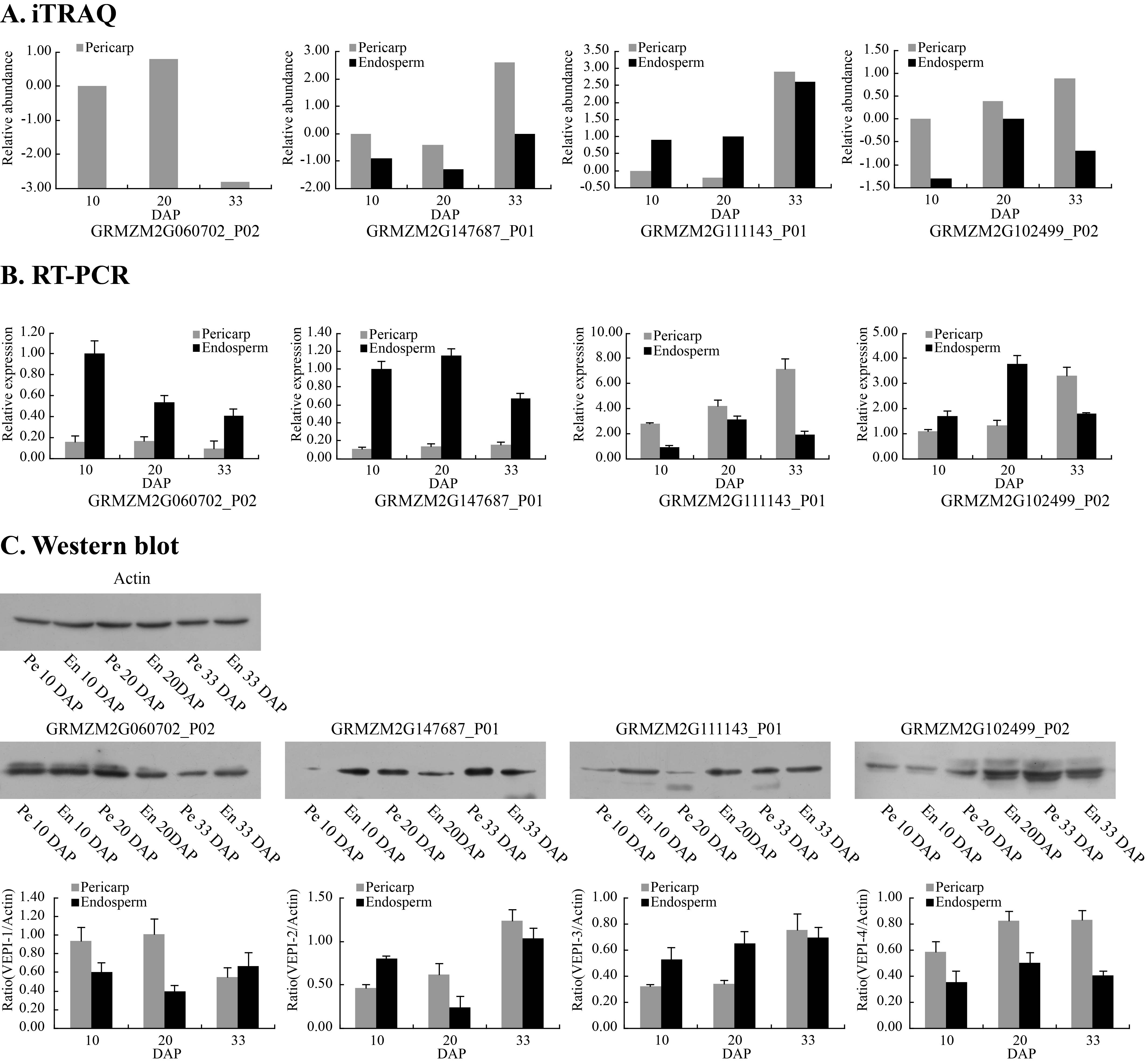


**Fig. C.** Real-time PCR and Western blot analysis of four proteins from iTRAQ in endosperm and pericarp at developmental stages for inbred line N04. (A) Relative abundance of the protein from iTRAQ, and all the groups were controled by pericarp at 10 DAP. (B) Real-time PCR for four proteins mRNA expression in IL N04 at 10 DAP, 20 DAP and 33 DAP. (C) Western blot analysis for the proteins after separation on SDS-PAGE, the proteins were controled by actin. VEPI, Validated the Expression of the Proteins by using iTRAQ. The results are presented as means+SEM pooled from three independent experiments in B and C.

**Table A. Specific proteins identified only in endosperm and pericarp for inbred line N04.**

Data are shown for specific proteins identified only in endosperm and pericarp for inbred line N04, specific proteins for pericarp and endosperm were 126 and 57, respectively.

| **Inbred** | **Tissue** | **Stage**  **(DAP)** | **Protein Accession** | **Functional-Categories** |
| --- | --- | --- | --- | --- |
| N04 | pericarp | 10 | GRMZM2G007647_P01 | Amino acid transport and metabolism |
| N04 | pericarp | 10 | GRMZM2G050371_P01 | Amino acid transport and metabolism ; |
| N04 | pericarp | 10 | GRMZM2G124365_P01 | Amino acid transport and metabolism ; |
| N04 | pericarp | 10 | AC182617.3_FGP001 | Amino acid transport and metabolism ; |
| N04 | pericarp | 10 | GRMZM2G573867_P01 | Amino acid transport and metabolism ; |
| N04 | pericarp | 10 | GRMZM2G386155_P01 | Amino acid transport and metabolism ; |
| N04 | pericarp | 10 | GRMZM2G076885_P01 | Amino acid transport and metabolism ; |
| N04 | pericarp | 10 | GRMZM2G068862_P01 | Amino acid transport and metabolism ; |
| N04 | pericarp | 10 | GRMZM2G068665_P01 | Amino acid transport and metabolism ; |
| N04 | pericarp | 10 | GRMZM2G058584_P01 | Amino acid transport and metabolism ; |
| N04 | pericarp | 10 | GRMZM5G836910_P01 | Amino acid transport and metabolism ; |
| N04 | pericarp | 10 | GRMZM2G143008_P01 | Amino acid transport and metabolism ; Coenzyme transport and metabolism |
| N04 | pericarp | 10 | GRMZM2G066290_P01 | Carbohydrate transport and metabolism |
| N04 | pericarp | 10 | GRMZM2G440208_P01 | Carbohydrate transport and metabolism |
| N04 | pericarp | 10 | GRMZM2G163437_P01 | Carbohydrate transport and metabolism |
| N04 | pericarp | 10 | GRMZM2G118462_P01 | Carbohydrate transport and metabolism |
| N04 | pericarp | 10 | GRMZM2G104081_P01 | Carbohydrate transport and metabolism |
| N04 | pericarp | 10 | GRMZM2G065083_P01 | Carbohydrate transport and metabolism |
| N04 | pericarp | 10 | GRMZM2G038821_P01 | Carbohydrate transport and metabolism ; Coenzyme transport and metabolism ; General function prediction only |
| N04 | pericarp | 10 | AC197705.4_FGP007 | Cell wall/membrane/envelope biogenesis |
| N04 | pericarp | 10 | GRMZM2G034069_P01 | Cell wall/membrane/envelope biogenesis ; Carbohydrate transport and metabolism |
| N04 | pericarp | 10 | GRMZM2G013821_P01 | Chromatin structure and dynamics |
| N04 | pericarp | 10 | GRMZM2G466833_P01 | Energy production and conversion |
| N04 | pericarp | 10 | GRMZM2G076239_P02 | Energy production and conversion |
| N04 | pericarp | 10 | GRMZM2G082581_P01 | Energy production and conversion |
| N04 | pericarp | 10 | GRMZM2G154595_P01 | Energy production and conversion |
| N04 | pericarp | 10 | GRMZM2G003789_P01 | General function prediction only |
| N04 | pericarp | 10 | GRMZM2G143330_P01 | General function prediction only |
| N04 | pericarp | 10 | GRMZM2G016827_P02 | General function prediction only |
| N04 | pericarp | 10 | GRMZM2G475293_P01 | General function prediction only |
| N04 | pericarp | 10 | GRMZM2G352129_P01 | General function prediction only |
| N04 | pericarp | 10 | GRMZM2G062425_P01 | General function prediction only |
| N04 | pericarp | 10 | GRMZM2G049866_P01 | General function prediction only |
| N04 | pericarp | 10 | GRMZM2G123234_P01 | General function prediction only |
| N04 | pericarp | 10 | GRMZM2G073700_P01 | General function prediction only |
| N04 | pericarp | 10 | GRMZM2G082198_P01 | General function prediction only |
| N04 | pericarp | 10 | GRMZM2G091819_P01 | Inorganic ion transport and metabolism |
| N04 | pericarp | 10 | GRMZM2G376731_P01 | Intracellular trafficking, secretion, and vesicular transport |
| N04 | pericarp | 10 | GRMZM2G004528_P03 | Lipid transport and metabolism |
| N04 | pericarp | 10 | GRMZM2G100084_P01 | Nucleotide transport and metabolism |
| N04 | pericarp | 10 | GRMZM2G044762_P01 | Nucleotide transport and metabolism |
| N04 | pericarp | 10 | GRMZM2G000622_P01 | Nucleotide transport and metabolism |
| N04 | pericarp | 10 | GRMZM2G013478_P01 | Nucleotide transport and metabolism |
| N04 | pericarp | 10 | GRMZM2G071846_P01 | Nucleotide transport and metabolism |
| N04 | pericarp | 10 | GRMZM2G105772_P01 | Posttranslational modification, protein turnover, chaperones |
| N04 | pericarp | 10 | GRMZM5G833699_P01 | Posttranslational modification, protein turnover, chaperones |
| N04 | pericarp | 10 | GRMZM2G083810_P01 | Posttranslational modification, protein turnover, chaperones |
| N04 | pericarp | 10 | GRMZM2G069651_P01 | Posttranslational modification, protein turnover, chaperones |
| N04 | pericarp | 10 | GRMZM2G038126_P01 | Posttranslational modification, protein turnover, chaperones |
| N04 | pericarp | 10 | GRMZM2G048277_P01 | Posttranslational modification, protein turnover, chaperones |
| N04 | pericarp | 10 | GRMZM2G329306_P01 | Posttranslational modification, protein turnover, chaperones |
| N04 | pericarp | 10 | AC215201.3_FGP005 | Posttranslational modification, protein turnover, chaperones |
| N04 | pericarp | 10 | GRMZM2G139680_P01 | Posttranslational modification, protein turnover, chaperones |
| N04 | pericarp | 10 | GRMZM2G002220_P01 | Posttranslational modification, protein turnover, chaperones |
| N04 | pericarp | 10 | GRMZM2G080724_P01 | Posttranslational modification, protein turnover, chaperones |
| N04 | pericarp | 10 | GRMZM2G028346_P01 | Posttranslational modification, protein turnover, chaperones |
| N04 | pericarp | 10 | GRMZM5G802801_P01 | Posttranslational modification, protein turnover, chaperones |
| N04 | pericarp | 10 | GRMZM2G047434_P01 | Posttranslational modification, protein turnover, chaperones |
| N04 | pericarp | 10 | GRMZM2G091189_P01 | Posttranslational modification, protein turnover, chaperones |
| N04 | pericarp | 10 | GRMZM2G587327_P01 | Posttranslational modification, protein turnover, chaperones |
| N04 | pericarp | 10 | GRMZM2G413652_P01 | Posttranslational modification, protein turnover, chaperones ; Transcription |
| N04 | pericarp | 10 | GRMZM2G014750_P01 | Posttranslational modification, protein turnover, chaperones ; Translation, ribosomal structure and biogenesis |
| N04 | pericarp | 10 | AC207890.3_FGP002 | Replication, recombination and repair |
| N04 | pericarp | 10 | GRMZM2G017532_P01 | Replication, recombination and repair ; General function prediction only |
| N04 | pericarp | 10 | GRMZM2G020142_P01 | Transcription |
| N04 | pericarp | 10 | AC194970.5_FGP009 | Transcription |
| N04 | pericarp | 10 | GRMZM2G083836_P01 | Translation, ribosomal structure and biogenesis |
| N04 | pericarp | 10 | GRMZM2G018943_P01 | Translation, ribosomal structure and biogenesis |
| N04 | pericarp | 10 | GRMZM2G125148_P01 | Translation, ribosomal structure and biogenesis |
| N04 | pericarp | 10 | GRMZM2G101463_P01 | Translation, ribosomal structure and biogenesis |
| N04 | pericarp | 10 | GRMZM2G125271_P01 | Translation, ribosomal structure and biogenesis |
| N04 | pericarp | 10 | GRMZM2G316232_P01 | Translation, ribosomal structure and biogenesis |
| N04 | pericarp | 10 | GRMZM2G069208_P01 | Function unknown |
| N04 | pericarp | 10 | GRMZM2G153162_P02 | Function unknown |
| N04 | pericarp | 10 | GRMZM2G375593_P01 | Function unknown |
| N04 | pericarp | 10 | GRMZM2G053019_P01 | Function unknown |
| N04 | pericarp | 10 | GRMZM2G172523_P01 | Function unknown |
| N04 | pericarp | 10 | GRMZM2G145226_P01 | Function unknown |
| N04 | pericarp | 10 | GRMZM2G056661_P01 | Function unknown |
| N04 | pericarp | 10 | GRMZM2G100146_P01 | Function unknown |
| N04 | pericarp | 10 | GRMZM2G034326_P01 | Function unknown |
| N04 | pericarp | 10 | GRMZM5G823017_P01 | Function unknown |
| N04 | pericarp | 10 | GRMZM2G097030_P01 | Function unknown |
| N04 | pericarp | 10 | GRMZM2G048194_P01 | Function unknown |
| N04 | pericarp | 10 | GRMZM5G888696_P02 | Function unknown |
| N04 | pericarp | 10 | GRMZM2G096806_P01 | Function unknown |
| N04 | pericarp | 10 | GRMZM2G704475_P02 | Function unknown |
| N04 | pericarp | 10 | GRMZM2G359397_P01 | Function unknown |
| N04 | pericarp | 10 | GRMZM2G161222_P03 | Function unknown |
| N04 | pericarp | 10 | GRMZM2G119627_P01 | Function unknown |
| N04 | pericarp | 10 | GRMZM2G031033_P01 | Function unknown |
| N04 | pericarp | 10 | GRMZM2G092627_P01 | Function unknown |
| N04 | pericarp | 10 | GRMZM2G164868_P01 | Function unknown |
| N04 | pericarp | 10 | GRMZM2G453424_P03 | Function unknown |
| N04 | pericarp | 10 | GRMZM2G412470_P02 | Function unknown |
| N04 | pericarp | 10 | GRMZM2G474575_P01 | Function unknown |
| N04 | pericarp | 10 | GRMZM2G353213_P01 | Function unknown |
| N04 | pericarp | 10 | GRMZM2G169182_P01 | Function unknown |
| N04 | pericarp | 10 | GRMZM2G047292_P01 | Function unknown |
| N04 | pericarp | 10 | GRMZM2G151387_P01 | Function unknown |
| N04 | pericarp | 10 | GRMZM2G347541_P01 | Function unknown |
| N04 | pericarp | 10 | GRMZM2G139341_P01 | Function unknown |
| N04 | pericarp | 10 | GRMZM2G098076_P01 | Function unknown |
| N04 | pericarp | 10 | GRMZM2G097207_P01 | Function unknown |
| N04 | pericarp | 10 | GRMZM2G480002_P01 | Function unknown |
| N04 | pericarp | 10 | GRMZM2G471357_P01 | Function unknown |
| N04 | pericarp | 10 | GRMZM2G101515_P01 | Function unknown |
| N04 | pericarp | 10 | GRMZM2G144081_P01 | Function unknown |
| N04 | pericarp | 10 | GRMZM2G353266_P01 | Function unknown |
| N04 | pericarp | 10 | GRMZM2G127160_P01 | Function unknown |
| N04 | pericarp | 10 | GRMZM2G167932_P01 | Function unknown |
| N04 | pericarp | 10 | GRMZM2G055434_P01 | Function unknown |
| N04 | pericarp | 10 | GRMZM2G376743_P01 | Function unknown |
| N04 | pericarp | 10 | GRMZM2G065292_P01 | Function unknown |
| N04 | pericarp | 20 | GRMZM2G163129_P01 | Carbohydrate transport and metabolism ; |
| N04 | pericarp | 20 | GRMZM2G068244_P01 | Cell wall/membrane/envelope biogenesis ; Carbohydrate transport and metabolism |
| N04 | pericarp | 20 | GRMZM2G036534_P01 | Energy production and conversion |
| N04 | pericarp | 20 | GRMZM2G170017_P01 | Lipid transport and metabolism ; Secondary metabolites biosynthesis, transport and catabolism ; General function prediction only |
| N04 | pericarp | 20 | GRMZM2G035017_P01 | Translation, ribosomal structure and biogenesis |
| N04 | pericarp | 20 | GRMZM2G112805_P01 | Function unknown |
| N04 | pericarp | 20 | GRMZM2G138727_P01 | Function unknown |
| N04 | pericarp | 20 | GRMZM2G107302_P01 | Function unknown |
| N04 | pericarp | 20 | GRMZM2G170969_P01 | Function unknown |
| N04 | pericarp | 33 | GRMZM2G068489_P01 | General function prediction only |
| N04 | pericarp | 33 | GRMZM2G044132_P01 | Function unknown |
| N04 | pericarp | 33 | AC155352.2_FGP010 | Function unknown |
| N04 | endosperm | 10 | GRMZM2G135283_P03 | Amino acid transport and metabolism |
| N04 | endosperm | 10 | GRMZM2G053999_P01 | Amino acid transport and metabolism |
| N04 | endosperm | 10 | GRMZM5G877500_P01 | Amino acid transport and metabolism |
| N04 | endosperm | 10 | GRMZM5G876898_P01 | Amino acid transport and metabolism |
| N04 | endosperm | 10 | GRMZM2G147221_P01 | Carbohydrate transport and metabolism |
| N04 | endosperm | 10 | AC197705.4_FGP007 | Cell wall/membrane/envelope biogenesis |
| N04 | endosperm | 10 | GRMZM2G143651_P01 | Cell wall/membrane/envelope biogenesis ; Carbohydrate transport and metabolism |
| N04 | endosperm | 10 | GRMZM2G117198_P01 | Coenzyme transport and metabolism |
| N04 | endosperm | 10 | GRMZM2G150485_P01 | Coenzyme transport and metabolism |
| N04 | endosperm | 10 | GRMZM2G133213_P01 | Coenzyme transport and metabolism ; Energy production and conversion |
| N04 | endosperm | 10 | AC234515.1_FGP003 | Cytoskeleton |
| N04 | endosperm | 10 | GRMZM2G030169_P01 | Cytoskeleton |
| N04 | endosperm | 10 | GRMZM2G051782_P01 | Cytoskeleton |
| N04 | endosperm | 10 | GRMZM2G156068_P01 | Energy production and conversion |
| N04 | endosperm | 10 | GRMZM2G154007_P01 | Energy production and conversion |
| N04 | endosperm | 10 | GRMZM2G003789_P01 | General function prediction only |
| N04 | endosperm | 10 | GRMZM2G061900_P01 | General function prediction only |
| N04 | endosperm | 10 | GRMZM2G018074_P01 | General function prediction only |
| N04 | endosperm | 10 | GRMZM2G055489_P01 | General function prediction only |
| N04 | endosperm | 10 | GRMZM2G129155_P01 | General function prediction only |
| N04 | endosperm | 10 | GRMZM2G335287_P01 | General function prediction only |
| N04 | endosperm | 10 | GRMZM2G005036_P01 | General function prediction only |
| N04 | endosperm | 10 | GRMZM2G026991_P01 | General function prediction only |
| N04 | endosperm | 10 | GRMZM5G827171_P01 | General function prediction only |
| N04 | endosperm | 10 | GRMZM2G320298_P01 | Lipid transport and metabolism |
| N04 | endosperm | 10 | GRMZM2G044947_P01 | Lipid transport and metabolism ; General function prediction only |
| N04 | endosperm | 10 | GRMZM2G146246_P02 | Posttranslational modification, protein turnover, chaperones |
| N04 | endosperm | 10 | GRMZM2G052435_P03 | Posttranslational modification, protein turnover, chaperones |
| N04 | endosperm | 10 | GRMZM2G056039_P01 | Posttranslational modification, protein turnover, chaperones |
| N04 | endosperm | 10 | GRMZM2G071441_P01 | Posttranslational modification, protein turnover, chaperones |
| N04 | endosperm | 10 | GRMZM2G112165_P01 | Posttranslational modification, protein turnover, chaperones |
| N04 | endosperm | 10 | AC192244.3_FGP001 | Posttranslational modification, protein turnover, chaperones |
| N04 | endosperm | 10 | GRMZM2G096705_P01 | Posttranslational modification, protein turnover, chaperones ; Translation, ribosomal structure and biogenesis |
| N04 | endosperm | 10 | GRMZM2G079817_P01 | Signal transduction mechanisms ; RNA processing and modification |
| N04 | endosperm | 10 | GRMZM2G171688_P01 | Translation, ribosomal structure and biogenesis |
| N04 | endosperm | 10 | GRMZM2G161274_P02 | Translation, ribosomal structure and biogenesis |
| N04 | endosperm | 10 | GRMZM2G035017_P01 | Translation, ribosomal structure and biogenesis |
| N04 | endosperm | 10 | GRMZM2G069208_P01 | Function unknown |
| N04 | endosperm | 10 | GRMZM2G047274_P01 | Function unknown |
| N04 | endosperm | 10 | GRMZM2G098496_P01 | Function unknown |
| N04 | endosperm | 10 | GRMZM2G047310_P02 | Function unknown |
| N04 | endosperm | 10 | GRMZM5G877388_P02 | Function unknown |
| N04 | endosperm | 10 | GRMZM2G133631_P01 | Function unknown |
| N04 | endosperm | 10 | GRMZM2G085967_P01 | Function unknown |
| N04 | endosperm | 10 | GRMZM2G108780_P01 | Function unknown |
| N04 | endosperm | 10 | GRMZM2G139341_P01 | Function unknown |
| N04 | endosperm | 10 | GRMZM2G151967_P01 | Function unknown |
| N04 | endosperm | 10 | GRMZM2G469111_P01 | Function unknown |
| N04 | endosperm | 20 | GRMZM2G071071_P01 | General function prediction only |
| N04 | endosperm | 33 | GRMZM2G006130_P01 | Carbohydrate transport and metabolism |
| N04 | endosperm | 33 | GRMZM2G056629_P01 | Carbohydrate transport and metabolism |
| N04 | endosperm | 33 | GRMZM2G473001_P01 | Energy production and conversion |
| N04 | endosperm | 33 | GRMZM2G139512_P01 | Energy production and conversion ; General function prediction only |
| N04 | endosperm | 33 | GRMZM2G079348_P01 | Inorganic ion transport and metabolism |
| N04 | endosperm | 33 | GRMZM2G073079_P01 | Secondary metabolites biosynthesis, transport and catabolism |
| N04 | endosperm | 33 | GRMZM5G825524_P01 | Function unknown |
| N04 | endosperm | 33 | GRMZM2G328171_P01 | Function unknown |

**Table B.** Protein functional-categories identified by Gene Ontology (GO) analysis for pericarp and endosperm of inbred line N04 at all developmental stages

| **Functional-Categories** | **N1** | **N2** | **N3** | **N4** | **N5** | **N6** | **Total** | **DEP** |
| --- | --- | --- | --- | --- | --- | --- | --- | --- |
| Amino acid transport and metabolism | 86 | 78 | 79 | 79 | 62 | 75 | 92 | 67 |
| Carbohydrate transport and metabolism | 86 | 86 | 84 | 82 | 75 | 83 | 97 | 63 |
| Cell cycle control acell division | 2 | 2 | 1 | 1 | 1 | 1 | 2 | 2 |
| Cell wall/membrane/envelope biogenesis | 27 | 27 | 25 | 18 | 14 | 15 | 30 | 24 |
| Chromatin structure and dynamics | 7 | 6 | 5 | 6 | 6 | 6 | 7 | 7 |
| Coenzyme transport and metabolism | 19 | 20 | 21 | 18 | 13 | 15 | 23 | 16 |
| Cytoskeleton | 14 | 14 | 13 | 14 | 11 | 11 | 15 | 11 |
| Energy production and conversion | 91 | 90 | 89 | 77 | 61 | 77 | 97 | 60 |
| General function | 112 | 109 | 108 | 108 | 91 | 96 | 126 | 86 |
| Inorganic ion transport and metabolism | 15 | 17 | 16 | 14 | 13 | 16 | 19 | 17 |
| Intracellular trafficking, secretion, and vesicular transport | 16 | 16 | 13 | 15 | 11 | 15 | 17 | 14 |
| Lipid transport and metabolism | 42 | 43 | 41 | 28 | 23 | 26 | 45 | 37 |
| Nuclear structure | 1 | 1 | 1 | 1 | 1 | 1 | 1 | 1 |
| Nucleotide transport and metabolism | 24 | 19 | 18 | 23 | 22 | 23 | 25 | 20 |
| Posttranslational modification | 162 | 146 | 143 | 160 | 148 | 151 | 172 | 132 |
| Replication, recombination and repair | 11 | 9 | 9 | 11 | 11 | 11 | 11 | 10 |
| RNA processing and modification | 1 | 1 | 1 | 1 | 1 | 1 | 2 | 2 |
| Secondary metabolites biosynthesis, transport and catabolism | 7 | 11 | 11 | 6 | 6 | 7 | 11 | 9 |
| Signal transduction mechanisms | 12 | 12 | 12 | 9 | 6 | 8 | 12 | 10 |
| Transcription | 8 | 6 | 6 | 6 | 5 | 5 | 8 | 7 |
| Translation, ribosomal structure and biogenesis | 104 | 100 | 94 | 106 | 100 | 99 | 108 | 87 |
| Function unknown | 398 | 410 | 392 | 356 | 325 | 339 | 481 | 386 |
| Total | 1245 | 1223 | 1182 | 1139 | 1006 | 1081 | 1396 | 1086 |
| DEP | 0 | 404 | 640 | 819 | 1062 | 876 | - | - |

Note: N1~N3 and N4~N6 represented pericarp and endosperm at 10 DAP, 20 DAP and 33DAP for inbred line N04, respectively; DEP differential expression proteins

**Table C.** Quantities of the differentially expressed proteins by contrast across different developmental stages and tissues.

| **Functional category** | **Stage comparison** | | | | **Tissue comparison** | | | |
| --- | --- | --- | --- | --- | --- | --- | --- | --- |
|  | **N2/N1** | **N3/N2** | **N5/N4** | **N6/N5** | | **N4/N1** | **N5/N2** | **N6/N3** |
| Amino acid transport and metabolism | 12/9 | 26/16 | 8/24 | 32/10 | | 21/44 | 20/43 | 22/37 |
| Carbohydrate transport and metabolism | 18/10 | 32/10 | 6/31 | 28/11 | | 34/45 | 26/39 | 29/41 |
| Cell cycle control, cell division, chromosome partitioning | 0/0 | 0/3 | 2/3 | 1/3 | | 1/4 | 2/2 | 1/1 |
| Cell wall/membrane/envelope biogenesis | 7/3 | 9/9 | 2/7 | 4/8 | | 5/11 | 6/12 | 2/8 |
| Chromatin structure and dynamics | 0/5 | 3/1 | 1/0 | 0/5 | | 4/1 | 3/1 | 1/1 |
| Coenzyme transport and metabolism | 2/2 | 7/6 | 4/11 | 5/5 | | 10/15 | 5/13 | 9/11 |
| Cytoskeleton | 0/8 | 1/7 | 3/5 | 1/10 | | 3/11 | 4/8 | 3/7 |
| Energy production and conversion | 9/13 | 33/32 | 26/29 | 37/18 | | 38/44 | 35/34 | 37/34 |
| General function prediction only | 13/27 | 32/44 | 30/20 | 17/57 | | 58/33 | 55/22 | 43/30 |
| Inorganic ion transport and metabolism | 0/1 | 0/6 | 0/1 | 0/10 | | 0/3 | 0/1 | 0/1 |
| Intracellular trafficking, secretion, and vesicular transport | 2/3 | 3/4 | 3/2 | 4/4 | | 4/10 | 5/7 | 5/9 |
| Lipid transport and metabolism | 3/7 | 11/11 | 3/11 | 7/5 | | 12/8 | 9/4 | 16/6 |
| Nuclear structure | 0/1 | 0/0 | 0/0 | 0/0 | | 0/1 | 0/0 | 0/1 |
| Nucleotide transport and metabolism | 4/1 | 7/4 | 5/3 | 9/6 | | 9/4 | 10/5 | 10/3 |
| Posttranslational modification, protein turnover, chaperones | 13/20 | 29/55 | 45/24 | 28/64 | | 40/75 | 44/58 | 38/49 |
| Replication, recombination and repair | 0/5 | 3/5 | 4/3 | 2/6 | | 6/4 | 6/4 | 5/3 |
| RNA processing and modification | 1/0 | 1/0 | 0/0 | 1/0 | | 2/0 | 1/0 | 1/0 |
| Secondary metabolites biosynthesis, transport and catabolism | 6/1 | 5/4 | 4/6 | 6/2 | | 4/6 | 5/2 | 3/4 |
| Signal transduction mechanisms | 1/3 | 4/6 | 4/1 | 0/6 | | 5/6 | 7/5 | 5/5 |
| Transcription | 0/5 | 4/6 | 4/3 | 1/9 | | 6/5 | 7/3 | 7/3 |
| Translation, ribosomal structure and biogenesis | 5/14 | 13/36 | 19/25 | 13/54 | | 41/48 | 38/33 | 21/39 |
| Function unknown | 72/88 | 129/113 | 112/34 | 55/163 | | 139/108 | 138/68 | 113/84 |
| Total | 168/226 | 352/378 | 285/243 | 251/456 | | 442/486 | 426/364 | 371/377 |

**Table D.** The functions of differentially expressed proteins integrated in the endosperm and pericarp networks

| **No.** | **Component** | **Differentially expressed proteins** | **Stage** | **First-degree Interaction protein** | **Function description** | **KEGG Orthology (KO)** | **KEGG Pathway Type** |
| --- | --- | --- | --- | --- | --- | --- | --- |
| 1 | Pericarp | GRMZM2G339994_P01 | N3; N3-N2 | ko00780_entry_67 | fabG; 3-oxoacyl-[acyl-carrier protein] reductase [EC:1.1.1.100] | ko:K00059 | Biotin metabolism |
|  |  |  |  | ko00780_entry_53 | fabG; 3-oxoacyl-[acyl-carrier protein] reductase [EC:1.1.1.100] | ko:K00059 | Biotin metabolism |
|  |  |  |  | ko00061_entry_91 | fabG; 3-oxoacyl-[acyl-carrier protein] reductase [EC:1.1.1.100] | ko:K00059 | Fatty acid biosynthesis |
|  |  |  |  | ko00061_entry_79 | fabG; 3-oxoacyl-[acyl-carrier protein] reductase [EC:1.1.1.100] | ko:K00059 | Fatty acid biosynthesis |
|  |  |  |  | ko00061_entry_54 | fabG; 3-oxoacyl-[acyl-carrier protein] reductase [EC:1.1.1.100] | ko:K00059 | Fatty acid biosynthesis |
|  |  |  |  | ko00061_entry_151 | fabG; 3-oxoacyl-[acyl-carrier protein] reductase [EC:1.1.1.100] | ko:K00059 | Fatty acid biosynthesis |
|  |  |  |  | ko00061_entry_139 | fabG; 3-oxoacyl-[acyl-carrier protein] reductase [EC:1.1.1.100] | ko:K00059 | Fatty acid biosynthesis |
|  |  |  |  | ko00061_entry_127 | fabG; 3-oxoacyl-[acyl-carrier protein] reductase [EC:1.1.1.100] | ko:K00059 | Fatty acid biosynthesis |
|  |  |  |  | ko00061_entry_115 | fabG; 3-oxoacyl-[acyl-carrier protein] reductase [EC:1.1.1.100] | ko:K00059 | Fatty acid biosynthesis |
|  |  |  |  | ko00061_entry_103 | fabG; 3-oxoacyl-[acyl-carrier protein] reductase [EC:1.1.1.100] | ko:K00059 | Fatty acid biosynthesis |
| 2 | Pericarp | GRMZM2G066202_P01 | N3 |  |  |  |  |
| 3 | Pericarp | GRMZM2G137535_P01 | N3 | ko00500_entry_97 | glucan endo-1,3-beta-D-glucosidase [EC:3.2.1.39]; E3.2.1.39 | ko:K01199 | Starch and sucrose metabolism |
| 4 | Pericarp | GRMZM2G005633_P02 | N3; N3-N2 | ko00520_entry_206 | bifunctional chitinase/lysozyme [EC:3.2.1.14 3.2.1.17]; chiA; E3.2.1.14; chitinase [EC:3.2.1.14] | ko:K01183 ko:K13381 | Amino sugar and nucleotide sugar metabolism |
|  |  |  |  | ko00520_entry_164 | bifunctional chitinase/lysozyme [EC:3.2.1.14 3.2.1.17]; chiA; E3.2.1.14; chitinase [EC:3.2.1.14] | ko:K01183 ko:K13381 | Amino sugar and nucleotide sugar metabolism |
| 5 | Pericarp | GRMZM2G080839_P01 | N3 |  |  |  |  |
| 6 | Pericarp | GRMZM2G094375_P01 | N3; N3-N2 | ko00910_entry_94 | E1.7.2.1; nitrite reductase (NO-forming) / hydroxylamine reductase [EC:1.7.2.1 1.7.99.1]; nirS; nitrite reductase (NO-forming) [EC:1.7.2.1] | ko:K00368 ko:K15864 | Nitrogen metabolism |
| 7 | Pericarp | GRMZM2G339091_P01 | N3 | ko00460_entry_51 | E4.1.2.11; hydroxymandelonitrile lyase [EC:4.1.2.11] | ko:K08249 | Cyanoamino acid metabolism |
| 8 | Pericarp | GRMZM2G403076_P01 | N3-N2 |  |  |  |  |
| 9 | Pericarp | GRMZM2G026703_P01 | N3 |  |  |  |  |
| 10 | Endosperm | GRMZM2G135283_P03 | N4 | ko00260_entry_85 | glyA, SHMT; glycine hydroxymethyltransferase [EC:2.1.2.1] | ko:K00600 | Glycine, serine and threonine metabolism |
|  |  |  |  | ko00460_entry_47 | glyA, SHMT; glycine hydroxymethyltransferase [EC:2.1.2.1] | ko:K00600 | Cyanoamino acid metabolism |
|  |  |  |  | ko00630_entry_216 | glyA, SHMT; glycine hydroxymethyltransferase [EC:2.1.2.1] | ko:K00600 | Glyoxylate and dicarboxylate metabolism |
|  |  |  |  | ko00670_entry_61 | glyA, SHMT; glycine hydroxymethyltransferase [EC:2.1.2.1] | ko:K00600 | One carbon pool by folate |
|  |  |  |  | ko00680_entry_311 | glyA, SHMT; glycine hydroxymethyltransferase [EC:2.1.2.1] | ko:K00600 | Methane metabolism |
|  |  |  |  | ko00680_entry_74 | glyA, SHMT; glycine hydroxymethyltransferase [EC:2.1.2.1] | ko:K00600 | Methane metabolism |
| 11 | Endosperm | GRMZM2G056431_P01 | N4; N5; N6 |  |  |  |  |
| 12 | Endosperm | GRMZM2G084149_P01 | N5; N6 |  |  |  |  |
| 13 | Endosperm | GRMZM2G008714_P01 | N4 | ko04930_entry_1 | PKLR; pyruvate kinase [EC:2.7.1.40]; pyruvate kinase isozymes R/L [EC:2.7.1.40]; PK, pyk | ko:K00873 ko:K12406 | Type II diabetes mellitus |
|  |  |  |  | ko00710_entry_59 | pyruvate kinase [EC:2.7.1.40]; PK, pyk | ko:K00873 | Carbon fixation in photosynthetic organisms |
|  |  |  |  | ko00620_entry_68 | PKLR; pyruvate kinase [EC:2.7.1.40]; pyruvate kinase isozymes R/L [EC:2.7.1.40]; PK, pyk | ko:K00873 ko:K12406 | Pyruvate metabolism |
|  |  |  |  | ko00230_entry_311 | PKLR; pyruvate kinase [EC:2.7.1.40]; pyruvate kinase isozymes R/L [EC:2.7.1.40]; PK, pyk | ko:K00873 ko:K12406 | Purine metabolism |
|  |  |  |  | ko00230_entry_310 | PKLR; pyruvate kinase [EC:2.7.1.40]; pyruvate kinase isozymes R/L [EC:2.7.1.40]; PK, pyk | ko:K00873 ko:K12406 | Purine metabolism |
|  |  |  |  | ko00230_entry_299 | PKLR; pyruvate kinase [EC:2.7.1.40]; pyruvate kinase isozymes R/L [EC:2.7.1.40]; PK, pyk | ko:K00873 ko:K12406 | Purine metabolism |
|  |  |  |  | ko00230_entry_298 | PKLR; pyruvate kinase [EC:2.7.1.40]; pyruvate kinase isozymes R/L [EC:2.7.1.40]; PK, pyk | ko:K00873 ko:K12406 | Purine metabolism |
|  |  |  |  | ko00010_entry_52 | PKLR; pyruvate kinase [EC:2.7.1.40]; pyruvate kinase isozymes R/L [EC:2.7.1.40]; PK, pyk | ko:K00873 ko:K12406 | Glycolysis / Gluconeogenesis |
| 14 | Endosperm | GRMZM2G039757_P01 | N4; N6 |  |  |  |  |
| 15 | Endosperm | GRMZM2G038821_P01 | N4; N5; N6 | ko00010_entry_46 | E4.1.1.1, pdc; pyruvate decarboxylase [EC:4.1.1.1] | ko:K01568 | Glycolysis / Gluconeogenesis |
|  |  |  |  | ko00010_entry_43 | E4.1.1.1, pdc; pyruvate decarboxylase [EC:4.1.1.1] | ko:K01568 | Glycolysis / Gluconeogenesis |
| 16 | Endosperm | GRMZM2G156785_P01 | N4; N5 |  |  |  |  |
| 17 | Endosperm | GRMZM2G130062_P01 | N4; N5; N6 | ko00620_entry_117 | 2-isopropylmalate synthase [EC:2.3.3.13]; leuA | ko:K01649 | Pyruvate metabolism |
|  |  |  |  | ko00290_entry_38 | 2-isopropylmalate synthase [EC:2.3.3.13]; leuA | ko:K01649 | Valine, leucine and isoleucine biosynthesis |
| 18 | Endosperm | GRMZM2G097226_P01 | N5 | ko00010_entry_47 | pyruvate dehydrogenase E1 component subunit beta [EC:1.2.4.1]; PDHA, pdhA; PDHB, pdhB; aceE; pyruvate dehydrogenase E1 component [EC:1.2.4.1]; pyruvate dehydrogenase E1 component subunit alpha [EC:1.2.4.1] | ko:K00163 ko:K00161 ko:K00162 | Glycolysis / Gluconeogenesis |
|  |  |  |  | ko00010_entry_48 | pyruvate dehydrogenase E1 component subunit beta [EC:1.2.4.1]; PDHA, pdhA; PDHB, pdhB; aceE; pyruvate dehydrogenase E1 component [EC:1.2.4.1]; pyruvate dehydrogenase E1 component subunit alpha [EC:1.2.4.1] | ko:K00163 ko:K00161 ko:K00162 | Glycolysis / Gluconeogenesis |
|  |  |  |  | ko00020_entry_79 | pyruvate dehydrogenase E1 component subunit beta [EC:1.2.4.1]; PDHA, pdhA; PDHB, pdhB; aceE; pyruvate dehydrogenase E1 component [EC:1.2.4.1]; pyruvate dehydrogenase E1 component subunit alpha [EC:1.2.4.1] | ko:K00163 ko:K00161 ko:K00162 | Citrate cycle (TCA cycle) |
|  |  |  |  | ko00020_entry_81 | pyruvate dehydrogenase E1 component subunit beta [EC:1.2.4.1]; PDHA, pdhA; PDHB, pdhB; aceE; pyruvate dehydrogenase E1 component [EC:1.2.4.1]; pyruvate dehydrogenase E1 component subunit alpha [EC:1.2.4.1] | ko:K00163 ko:K00161 ko:K00162 | Citrate cycle (TCA cycle) |
|  |  |  |  | ko00620_entry_131 | pyruvate dehydrogenase E1 component subunit beta [EC:1.2.4.1]; PDHA, pdhA; PDHB, pdhB; aceE; pyruvate dehydrogenase E1 component [EC:1.2.4.1]; pyruvate dehydrogenase E1 component subunit alpha [EC:1.2.4.1] | ko:K00163 ko:K00161 ko:K00162 | Pyruvate metabolism |
|  |  |  |  | ko00620_entry_58 | pyruvate dehydrogenase E1 component subunit beta [EC:1.2.4.1]; PDHA, pdhA; PDHB, pdhB; aceE; pyruvate dehydrogenase E1 component [EC:1.2.4.1]; pyruvate dehydrogenase E1 component subunit alpha [EC:1.2.4.1] | ko:K00163 ko:K00161 ko:K00162 | Pyruvate metabolism |
|  |  |  |  | ko04066_entry_37 | pyruvate dehydrogenase E1 component subunit beta [EC:1.2.4.1]; PDHB, pdhB; pyruvate dehydrogenase E1 component subunit alpha [EC:1.2.4.1]; PDHA, pdhA | ko:K00161 ko:K00162 | HIF-1 signaling pathway |
| 19 | Endosperm | GRMZM2G137329_P01 | N6; N6-N4 |  |  |  |  |
| 20 | Endosperm | GRMZM2G419675_P01 | N6; N6-N4 |  |  |  |  |
| 21 | Endosperm | GRMZM2G154595_P01 | N5 | ko00710_entry_81 | mdh; malate dehydrogenase [EC:1.1.1.37]; MDH1; MDH2 | ko:K00025 ko:K00026 ko:K00024 | Carbon fixation in photosynthetic organisms |
|  |  |  |  | ko00710_entry_60 | mdh; malate dehydrogenase [EC:1.1.1.37]; MDH1; MDH2 | ko:K00025 ko:K00026 ko:K00024 | Carbon fixation in photosynthetic organisms |
|  |  |  |  | ko00630_entry_123 | mdh; malate dehydrogenase [EC:1.1.1.37]; MDH1; MDH2 | ko:K00025 ko:K00026 ko:K00024 | Glyoxylate and dicarboxylate metabolism |
|  |  |  |  | ko00620_entry_93 | mdh; malate dehydrogenase [EC:1.1.1.37]; MDH1; MDH2 | ko:K00025 ko:K00026 ko:K00024 | Pyruvate metabolism |
|  |  |  |  | ko00020_entry_44 | mdh; malate dehydrogenase [EC:1.1.1.37]; MDH1; MDH2 | ko:K00025 ko:K00026 ko:K00024 | Citrate cycle (TCA cycle) |
| 22 | Endosperm | GRMZM2G147221_P01 | N5 | ko00500_entry_158 | E3.2.1.58; glucan 1,3-beta-glucosidase [EC:3.2.1.58] | ko:K01210 | Starch and sucrose metabolism |
| 23 | Endosperm | GRMZM2G074158_P01 | N4; N5; N6 | ko04910_entry_27 | starch phosphorylase [EC:2.4.1.1]; E2.4.1.1, glgP, PYG | ko:K00688 | Insulin signaling pathway |
|  |  |  |  | ko00500_entry_150 | K16153; phosphorylase / glycogen(starch) synthase [EC:2.4.1.1 2.4.1.11]; starch phosphorylase [EC:2.4.1.1]; E2.4.1.1, glgP, PYG | ko:K00688 ko:K16153 | Starch and sucrose metabolism |
| 24 | Endosperm | GRMZM2G158043_P02 | N4; N5 | ko00500_entry_250 | treY, glgY; treX, glgX; maltooligosyltrehalose trehalohydrolase [EC:3.2.1.141]; treZ, glgZ; (1->4)-alpha-D-glucan 1-alpha-D-glucosylmutase [EC:5.4.99.15]; glycogen operon protein [EC:3.2.1.-] | ko:K02438 ko:K06044 ko:K01236 | Starch and sucrose metabolism |
| 25 | Endosperm | GRMZM2G014376_P01 | N5; N6 | ko00400_entry_75 | ARO1; pentafunctional AROM polypeptide [EC:4.2.3.4 4.2.1.10 1.1.1.25 2.7.1.71 2.5.1.19]; 3-dehydroquinate dehydratase I [EC:4.2.1.10]; aroQ, qutE; 3-dehydroquinate dehydratase / shikimate dehydrogenase [EC:4.2.1.10 1.1.1.25]; aroD; aroDE, DHQ-SDH; 3-dehydroquinate dehydratase II [EC:4.2.1.10] | ko:K03785 ko:K03786 ko:K13832 ko:K13830 | Phenylalanine, tyrosine and tryptophan biosynthesis |
|  |  |  |  | ko00400_entry_74 | ARO1; pentafunctional AROM polypeptide [EC:4.2.3.4 4.2.1.10 1.1.1.25 2.7.1.71 2.5.1.19]; 3-dehydroquinate dehydratase / shikimate dehydrogenase [EC:4.2.1.10 1.1.1.25]; shikimate dehydrogenase [EC:1.1.1.25]; aroDE, DHQ-SDH; aroE | ko:K00014 ko:K13832 ko:K13830 | Phenylalanine, tyrosine and tryptophan biosynthesis |
| 26 | Endosperm | GRMZM2G047292_P01 | N4; N5; N6 | ko00052_entry_62 | E2.4.1.67; stachyose synthetase [EC:2.4.1.67] | ko:K06611 | Galactose metabolism |
| 27 | Endosperm | GRMZM2G101408_P01 | N5 |  |  |  |  |
| 28 | Endosperm | GRMZM2G120652_P01 | N5 | ko00750_entry_4 | pdxS, pdx1; pyridoxine biosynthesis protein [EC:4.-.-.-] | ko:K06215 | Vitamin B6 metabolism |
| 29 | Endosperm | GRMZM5G881950_P01 | N4; N5; N6 |  |  |  |  |
| 30 | Endosperm | GRMZM2G090087_P02 | N6 | ko00250_entry_115 | putA; E1.5.1.12; proline dehydrogenase / delta 1-pyrroline-5-carboxylate dehydrogenase [EC:1.5.99.8 1.5.1.12]; 1-pyrroline-5-carboxylate dehydrogenase [EC:1.5.1.12] | ko:K00294 ko:K13821 | Alanine, aspartate and glutamate metabolism |
|  |  |  |  | ko00330_entry_199 | putA; E1.5.1.12; proline dehydrogenase / delta 1-pyrroline-5-carboxylate dehydrogenase [EC:1.5.99.8 1.5.1.12]; 1-pyrroline-5-carboxylate dehydrogenase [EC:1.5.1.12] | ko:K00294 ko:K13821 | Arginine and proline metabolism |
|  |  |  |  | ko00330_entry_200 | putA; E1.5.1.12; proline dehydrogenase / delta 1-pyrroline-5-carboxylate dehydrogenase [EC:1.5.99.8 1.5.1.12]; 1-pyrroline-5-carboxylate dehydrogenase [EC:1.5.1.12] | ko:K00294 ko:K13821 | Arginine and proline metabolism |
|  |  |  |  | ko00330_entry_238 | putA; E1.5.1.12; proline dehydrogenase / delta 1-pyrroline-5-carboxylate dehydrogenase [EC:1.5.99.8 1.5.1.12]; 1-pyrroline-5-carboxylate dehydrogenase [EC:1.5.1.12] | ko:K00294 ko:K13821 | Arginine and proline metabolism |
|  |  |  |  | ko00330_entry_308 | putA; E1.5.1.12; proline dehydrogenase / delta 1-pyrroline-5-carboxylate dehydrogenase [EC:1.5.99.8 1.5.1.12]; 1-pyrroline-5-carboxylate dehydrogenase [EC:1.5.1.12] | ko:K00294 ko:K13821 | Arginine and proline metabolism |
| 31 | Endosperm | GRMZM2G143862_P01 | N4; N5 | ko00051_entry_96 | E2.7.1.90, pfk; pyrophosphate--fructose-6-phosphate 1-phosphotransferase [EC:2.7.1.90] | ko:K00895 | Fructose and mannose metabolism |
| 32 | Endosperm | GRMZM2G149717_P01 | N4; N6 | ko05100_entry_33 | DNM; dynamin GTPase [EC:3.6.5.5] | ko:K01528 | Bacterial invasion of epithelial cells |
| 33 | Endosperm | GRMZM2G328171_P01 | N6 | ko00520_entry_164 | bifunctional chitinase/lysozyme [EC:3.2.1.14 3.2.1.17]; chiA; E3.2.1.14; chitinase [EC:3.2.1.14] | ko:K01183 ko:K13381 | Amino sugar and nucleotide sugar metabolism |
|  |  |  |  | ko00520_entry_206 | bifunctional chitinase/lysozyme [EC:3.2.1.14 3.2.1.17]; chiA; E3.2.1.14; chitinase [EC:3.2.1.14] | ko:K01183 ko:K13381 | Amino sugar and nucleotide sugar metabolism |
| 34 | Endosperm | GRMZM2G447795_P01 | N6 | ko00520_entry_206 | bifunctional chitinase/lysozyme [EC:3.2.1.14 3.2.1.17]; chiA; E3.2.1.14; chitinase [EC:3.2.1.14] | ko:K01183 ko:K13381 | Amino sugar and nucleotide sugar metabolism |
|  |  |  |  | ko00520_entry_164 | bifunctional chitinase/lysozyme [EC:3.2.1.14 3.2.1.17]; chiA; E3.2.1.14; chitinase [EC:3.2.1.14] | ko:K01183 ko:K13381 | Amino sugar and nucleotide sugar metabolism |
| 35 | Endosperm | GRMZM2G005633_P02 | N6 | ko00520_entry_164 | bifunctional chitinase/lysozyme [EC:3.2.1.14 3.2.1.17]; chiA; E3.2.1.14; chitinase [EC:3.2.1.14] | ko:K01183 ko:K13381 | Amino sugar and nucleotide sugar metabolism |
|  |  |  |  | ko00520_entry_206 | bifunctional chitinase/lysozyme [EC:3.2.1.14 3.2.1.17]; chiA; E3.2.1.14; chitinase [EC:3.2.1.14] | ko:K01183 ko:K13381 | Amino sugar and nucleotide sugar metabolism |
| 36 | Endosperm | GRMZM2G328893_P01 | N4; N6 | ko00920_entry_49 | cysM; cysO; cysK; cysteine synthase / O-phosphoserine sulfhydrylase / spermine synthase [EC:2.5.1.47 2.5.1.65 4.2.1.22]; ATCYSC1; cysteine synthase A [EC:2.5.1.47]; L-3-cyanoalanine synthase/ cysteine synthase [EC:2.5.1.47 4.4.1.9]; cysteine synthase B [EC:2.5.1.47] | ko:K01738 ko:K12339 ko:K13034 ko:K10150 | Sulfur metabolism |
|  |  |  |  | ko00270_entry_126 | cysM; cysO; cysK; MET17; cysteine synthase / O-phosphoserine sulfhydrylase / spermine synthase [EC:2.5.1.47 2.5.1.65 4.2.1.22]; ATCYSC1; cysteine synthase A [EC:2.5.1.47]; L-3-cyanoalanine synthase/ cysteine synthase [EC:2.5.1.47 4.4.1.9]; cysteine synthase B [EC:2.5.1.47]; O-acetylhomoserine/O-acetylserine sulfhydrylase [EC:2.5.1.49 2.5.1.47] | ko:K01738 ko:K12339 ko:K13034 ko:K10150 ko:K17069 | Cysteine and methionine metabolism |
|  |  |  |  | ko00270_entry_119 | cysM; cysO; cysK; MET17; cysteine synthase / O-phosphoserine sulfhydrylase / spermine synthase [EC:2.5.1.47 2.5.1.65 4.2.1.22]; ATCYSC1; cysteine synthase A [EC:2.5.1.47]; L-3-cyanoalanine synthase/ cysteine synthase [EC:2.5.1.47 4.4.1.9]; cysteine synthase B [EC:2.5.1.47]; O-acetylhomoserine/O-acetylserine sulfhydrylase [EC:2.5.1.49 2.5.1.47] | ko:K01738 ko:K12339 ko:K13034 ko:K10150 ko:K17069 | Cysteine and methionine metabolism |
| 37 | Endosperm | GRMZM2G054123_P01 | N6 | ko00270_entry_88 | metK; S-adenosylmethionine synthetase [EC:2.5.1.6] | ko:K00789 | Cysteine and methionine metabolism |
| 38 | Endosperm | GRMZM2G117198_P01 | N4 | ko00270_entry_88 | metK; S-adenosylmethionine synthetase [EC:2.5.1.6] | ko:K00789 | Cysteine and methionine metabolism |
| 39 | Endosperm | GRMZM2G339994_P01 | N6 | ko00061_entry_103 | fabG; 3-oxoacyl-[acyl-carrier protein] reductase [EC:1.1.1.100] | ko:K00059 | Fatty acid biosynthesis |
|  |  |  |  | ko00061_entry_115 | fabG; 3-oxoacyl-[acyl-carrier protein] reductase [EC:1.1.1.100] | ko:K00059 | Fatty acid biosynthesis |
|  |  |  |  | ko00061_entry_127 | fabG; 3-oxoacyl-[acyl-carrier protein] reductase [EC:1.1.1.100] | ko:K00059 | Fatty acid biosynthesis |
|  |  |  |  | ko00061_entry_139 | fabG; 3-oxoacyl-[acyl-carrier protein] reductase [EC:1.1.1.100] | ko:K00059 | Fatty acid biosynthesis |
|  |  |  |  | ko00061_entry_151 | fabG; 3-oxoacyl-[acyl-carrier protein] reductase [EC:1.1.1.100] | ko:K00059 | Fatty acid biosynthesis |
|  |  |  |  | ko00061_entry_54 | fabG; 3-oxoacyl-[acyl-carrier protein] reductase [EC:1.1.1.100] | ko:K00059 | Fatty acid biosynthesis |
|  |  |  |  | ko00061_entry_79 | fabG; 3-oxoacyl-[acyl-carrier protein] reductase [EC:1.1.1.100] | ko:K00059 | Fatty acid biosynthesis |
|  |  |  |  | ko00061_entry_91 | fabG; 3-oxoacyl-[acyl-carrier protein] reductase [EC:1.1.1.100] | ko:K00059 | Fatty acid biosynthesis |
|  |  |  |  | ko00780_entry_53 | fabG; 3-oxoacyl-[acyl-carrier protein] reductase [EC:1.1.1.100] | ko:K00059 | Biotin metabolism |
|  |  |  |  | ko00780_entry_67 | fabG; 3-oxoacyl-[acyl-carrier protein] reductase [EC:1.1.1.100] | ko:K00059 | Biotin metabolism |
| 40 | Endosperm | GRMZM2G157329_P01 | N4; N5; N6 | ko03015_entry_12 | THOC4, ALY; THO complex subunit 4 | ko:K12881 | mRNA surveillance pathway |
|  |  |  |  | ko03013_entry_34 | THOC4, ALY; THO complex subunit 4 | ko:K12881 | RNA transport |
| 41 | Endosperm | GRMZM2G018947_P01 | N4; N5; N6 | ko03013_entry_25 | ATP-dependent RNA helicase [EC:3.6.4.13]; EIF4A3, FAL1 | ko:K13025 | RNA transport |
|  |  |  |  | ko03015_entry_4 | ATP-dependent RNA helicase [EC:3.6.4.13]; EIF4A3, FAL1 | ko:K13025 | mRNA surveillance pathway |
| 42 | Endosperm | GRMZM2G134107_P01 | N5 |  |  |  |  |
| 43 | Endosperm | GRMZM2G445905_P01 | N6 | ko03013_entry_96 | EIF4E; translation initiation factor 4E | ko:K03259 | RNA transport |
|  |  |  |  | ko04066_entry_81 | EIF4E; translation initiation factor 4E | ko:K03259 | HIF-1 signaling pathway |
|  |  |  |  | ko04150_entry_15 | EIF4E; translation initiation factor 4E | ko:K03259 | mTOR signaling pathway |
|  |  |  |  | ko04151_entry_2 | EIF4E; translation initiation factor 4E | ko:K03259 | PI3K-Akt signaling pathway |
|  |  |  |  | ko04910_entry_32 | EIF4E; translation initiation factor 4E | ko:K03259 | Insulin signaling pathway |
| 44 | Endosperm | GRMZM2G001898_P01 | N4; N5; N6 | ko00640_entry_86 | malonate-semialdehyde dehydrogenase (acetylating) / methylmalonate-semialdehyde dehydrogenase [EC:1.2.1.18 1.2.1.27]; mmsA, iolA, ALDH6A1 | ko:K00140 | Propanoate metabolism |
|  |  |  |  | ko00640_entry_55 | malonate-semialdehyde dehydrogenase (acetylating) / methylmalonate-semialdehyde dehydrogenase [EC:1.2.1.18 1.2.1.27]; mmsA, iolA, ALDH6A1 | ko:K00140 | Propanoate metabolism |
|  |  |  |  | ko00640_entry_54 | malonate-semialdehyde dehydrogenase (acetylating) / methylmalonate-semialdehyde dehydrogenase [EC:1.2.1.18 1.2.1.27]; mmsA, iolA, ALDH6A1 | ko:K00140 | Propanoate metabolism |
|  |  |  |  | ko00640_entry_106 | malonate-semialdehyde dehydrogenase (acetylating) / methylmalonate-semialdehyde dehydrogenase [EC:1.2.1.18 1.2.1.27]; mmsA, iolA, ALDH6A1 | ko:K00140 | Propanoate metabolism |
|  |  |  |  | ko00562_entry_127 | malonate-semialdehyde dehydrogenase (acetylating) / methylmalonate-semialdehyde dehydrogenase [EC:1.2.1.18 1.2.1.27]; mmsA, iolA, ALDH6A1 | ko:K00140 | Inositol phosphate metabolism |
|  |  |  |  | ko00410_entry_45 | malonate-semialdehyde dehydrogenase (acetylating) / methylmalonate-semialdehyde dehydrogenase [EC:1.2.1.18 1.2.1.27]; mmsA, iolA, ALDH6A1 | ko:K00140 | beta-Alanine metabolism |
|  |  |  |  | ko00280_entry_102 | malonate-semialdehyde dehydrogenase (acetylating) / methylmalonate-semialdehyde dehydrogenase [EC:1.2.1.18 1.2.1.27]; mmsA, iolA, ALDH6A1 | ko:K00140 | Valine, leucine and isoleucine degradation |
| 45 | Endosperm | GRMZM2G021219_P01 | N4; N5 |  |  |  |  |
| 46 | Endosperm | GRMZM2G134889_P01 | N6 | ko04141_entry_77 | PDIA1, P4HB; protein disulfide-isomerase A6 [EC:5.3.4.1]; ERP29; protein disulfide-isomerase [EC:5.3.4.1]; thioredoxin domain-containing protein 5; PDIA4, ERP72; protein disulfide-isomerase A4 [EC:5.3.4.1]; endoplasmic reticulum protein 29; TXNDC5, ERP46; PDIA6, TXNDC7; protein disulfide-isomerase A1 [EC:5.3.4.1]; EPS1 | ko:K09580 ko:K09582 ko:K09584 ko:K13996 ko:K09586 ko:K13984 | Protein processing in endoplasmic reticulum |
| 47 | Endosperm | GRMZM2G022269_P01 | N4; N6 | ko04626_entry_4 | elongation factor Tu; tuf, TUFM | ko:K02358 | Plant-pathogen interaction |
| 48 | Endosperm | GRMZM2G097030_P01 | N4; N5; N6 | ko00402_entry_21 | UDP-glucosyltransferase BX8 [EC:2.4.1.202]; BX8; UDP-glucosyltransferase BX9 [EC:2.4.1.202]; BX9 | ko:K13227 ko:K13228 | Benzoxazinoid biosynthesis |
| 49 | Endosperm | GRMZM2G145968_P01 | N4; N5; N6 |  |  |  |  |
| 50 | Endosperm | GRMZM2G132903_P01 | N4; N5; N6 | ko00592_entry_26 | enoyl-CoA hydratase/3-hydroxyacyl-CoA dehydrogenase [EC:4.2.1.17 1.1.1.35 1.1.1.211]; MFP2 | ko:K10527 | alpha-Linolenic acid metabolism |
|  |  |  |  | ko00592_entry_25 | enoyl-CoA hydratase/3-hydroxyacyl-CoA dehydrogenase [EC:4.2.1.17 1.1.1.35 1.1.1.211]; MFP2 | ko:K10527 | alpha-Linolenic acid metabolism |
|  |  |  |  | ko00592_entry_24 | enoyl-CoA hydratase/3-hydroxyacyl-CoA dehydrogenase [EC:4.2.1.17 1.1.1.35 1.1.1.211]; MFP2 | ko:K10527 | alpha-Linolenic acid metabolism |
|  |  |  |  | ko00071_entry_97 | enoyl-CoA hydratase / long-chain 3-hydroxyacyl-CoA dehydrogenase [EC:4.2.1.17 1.1.1.211]; HADHA; enoyl-CoA hydratase/3-hydroxyacyl-CoA dehydrogenase [EC:4.2.1.17 1.1.1.35 1.1.1.211]; MFP2 | ko:K07515 ko:K10527 | Fatty acid metabolism |
|  |  |  |  | ko00071_entry_93 | HADH; 3-hydroxyacyl-CoA dehydrogenase [EC:1.1.1.35]; enoyl-CoA hydratase / 3-hydroxyacyl-CoA dehydrogenase / 3,2-trans-enoyl-CoA isomerase [EC:4.2.1.17 1.1.1.35 5.3.3.8]; enoyl-CoA hydratase/3-hydroxyacyl-CoA dehydrogenase [EC:4.2.1.17 1.1.1.35 1.1.1.211]; 3-hydroxyacyl-CoA dehydrogenase / enoyl-CoA hydratase / 3-hydroxybutyryl-CoA epimerase [EC:1.1.1.35 4.2.1.17 5.1.2.3]; fadN; fadB; fadJ; MFP2; EHHADH; 3-hydroxyacyl-CoA dehydrogenase / enoyl-CoA hydratase / 3-hydroxybutyryl-CoA epimerase / enoyl-CoA isomerase [EC:1.1.1.35 4.2.1.17 5.1.2.3 5.3.3.8] | ko:K00022 ko:K07516 ko:K10527 ko:K07514 ko:K01825 ko:K01782 | Fatty acid metabolism |
|  |  |  |  | ko00071_entry_91 | enoyl-CoA hydratase / 3-hydroxyacyl-CoA dehydrogenase / 3,2-trans-enoyl-CoA isomerase [EC:4.2.1.17 1.1.1.35 5.3.3.8]; enoyl-CoA hydratase/3-hydroxyacyl-CoA dehydrogenase [EC:4.2.1.17 1.1.1.35 1.1.1.211]; ECHS1; HADHA; 3-hydroxyacyl-CoA dehydrogenase / enoyl-CoA hydratase / 3-hydroxybutyryl-CoA epimerase [EC:1.1.1.35 4.2.1.17 5.1.2.3]; ysiB, fadB; MFP2; fadB; enoyl-CoA hydratase / long-chain 3-hydroxyacyl-CoA dehydrogenase [EC:4.2.1.17 1.1.1.211]; fadJ; enoyl-CoA hydratase [EC:4.2.1.17]; paaF, echA; EHHADH; 3-hydroxyacyl-CoA dehydrogenase / enoyl-CoA hydratase / 3-hydroxybutyryl-CoA epimerase / enoyl-CoA isomerase [EC:1.1.1.35 4.2.1.17 5.1.2.3 5.3.3.8] | ko:K01692 ko:K10527 ko:K01825 ko:K01782 ko:K07511 ko:K13767 ko:K07514 ko:K07515 | Fatty acid metabolism |
|  |  |  |  | ko00071_entry_84 | enoyl-CoA hydratase / long-chain 3-hydroxyacyl-CoA dehydrogenase [EC:4.2.1.17 1.1.1.211]; HADHA; enoyl-CoA hydratase/3-hydroxyacyl-CoA dehydrogenase [EC:4.2.1.17 1.1.1.35 1.1.1.211]; MFP2 | ko:K07515 ko:K10527 | Fatty acid metabolism |
|  |  |  |  | ko00071_entry_77 | enoyl-CoA hydratase / 3-hydroxyacyl-CoA dehydrogenase / 3,2-trans-enoyl-CoA isomerase [EC:4.2.1.17 1.1.1.35 5.3.3.8]; enoyl-CoA hydratase/3-hydroxyacyl-CoA dehydrogenase [EC:4.2.1.17 1.1.1.35 1.1.1.211]; ECHS1; HADHA; 3-hydroxyacyl-CoA dehydrogenase / enoyl-CoA hydratase / 3-hydroxybutyryl-CoA epimerase [EC:1.1.1.35 4.2.1.17 5.1.2.3]; ysiB, fadB; MFP2; fadB; enoyl-CoA hydratase / long-chain 3-hydroxyacyl-CoA dehydrogenase [EC:4.2.1.17 1.1.1.211]; fadJ; enoyl-CoA hydratase [EC:4.2.1.17]; paaF, echA; EHHADH; 3-hydroxyacyl-CoA dehydrogenase / enoyl-CoA hydratase / 3-hydroxybutyryl-CoA epimerase / enoyl-CoA isomerase [EC:1.1.1.35 4.2.1.17 5.1.2.3 5.3.3.8] | ko:K01692 ko:K10527 ko:K01825 ko:K01782 ko:K07511 ko:K13767 ko:K07514 ko:K07515 | Fatty acid metabolism |
|  |  |  |  | ko00071_entry_76 | HADH; 3-hydroxyacyl-CoA dehydrogenase [EC:1.1.1.35]; enoyl-CoA hydratase / 3-hydroxyacyl-CoA dehydrogenase / 3,2-trans-enoyl-CoA isomerase [EC:4.2.1.17 1.1.1.35 5.3.3.8]; enoyl-CoA hydratase/3-hydroxyacyl-CoA dehydrogenase [EC:4.2.1.17 1.1.1.35 1.1.1.211]; 3-hydroxyacyl-CoA dehydrogenase / enoyl-CoA hydratase / 3-hydroxybutyryl-CoA epimerase [EC:1.1.1.35 4.2.1.17 5.1.2.3]; fadN; fadB; fadJ; MFP2; EHHADH; 3-hydroxyacyl-CoA dehydrogenase / enoyl-CoA hydratase / 3-hydroxybutyryl-CoA epimerase / enoyl-CoA isomerase [EC:1.1.1.35 4.2.1.17 5.1.2.3 5.3.3.8] | ko:K00022 ko:K07516 ko:K10527 ko:K07514 ko:K01825 ko:K01782 | Fatty acid metabolism |
|  |  |  |  | ko00071_entry_70 | enoyl-CoA hydratase / long-chain 3-hydroxyacyl-CoA dehydrogenase [EC:4.2.1.17 1.1.1.211]; HADHA; enoyl-CoA hydratase/3-hydroxyacyl-CoA dehydrogenase [EC:4.2.1.17 1.1.1.35 1.1.1.211]; MFP2 | ko:K07515 ko:K10527 | Fatty acid metabolism |
|  |  |  |  | ko00071_entry_67 | enoyl-CoA hydratase / 3-hydroxyacyl-CoA dehydrogenase / 3,2-trans-enoyl-CoA isomerase [EC:4.2.1.17 1.1.1.35 5.3.3.8]; enoyl-CoA hydratase/3-hydroxyacyl-CoA dehydrogenase [EC:4.2.1.17 1.1.1.35 1.1.1.211]; ECHS1; HADHA; 3-hydroxyacyl-CoA dehydrogenase / enoyl-CoA hydratase / 3-hydroxybutyryl-CoA epimerase [EC:1.1.1.35 4.2.1.17 5.1.2.3]; ysiB, fadB; MFP2; fadB; enoyl-CoA hydratase / long-chain 3-hydroxyacyl-CoA dehydrogenase [EC:4.2.1.17 1.1.1.211]; fadJ; enoyl-CoA hydratase [EC:4.2.1.17]; paaF, echA; EHHADH; 3-hydroxyacyl-CoA dehydrogenase / enoyl-CoA hydratase / 3-hydroxybutyryl-CoA epimerase / enoyl-CoA isomerase [EC:1.1.1.35 4.2.1.17 5.1.2.3 5.3.3.8] | ko:K01692 ko:K10527 ko:K01825 ko:K01782 ko:K07511 ko:K13767 ko:K07514 ko:K07515 | Fatty acid metabolism |
|  |  |  |  | ko00071_entry_66 | HADH; 3-hydroxyacyl-CoA dehydrogenase [EC:1.1.1.35]; enoyl-CoA hydratase / 3-hydroxyacyl-CoA dehydrogenase / 3,2-trans-enoyl-CoA isomerase [EC:4.2.1.17 1.1.1.35 5.3.3.8]; enoyl-CoA hydratase/3-hydroxyacyl-CoA dehydrogenase [EC:4.2.1.17 1.1.1.35 1.1.1.211]; 3-hydroxyacyl-CoA dehydrogenase / enoyl-CoA hydratase / 3-hydroxybutyryl-CoA epimerase [EC:1.1.1.35 4.2.1.17 5.1.2.3]; fadN; fadB; fadJ; MFP2; EHHADH; 3-hydroxyacyl-CoA dehydrogenase / enoyl-CoA hydratase / 3-hydroxybutyryl-CoA epimerase / enoyl-CoA isomerase [EC:1.1.1.35 4.2.1.17 5.1.2.3 5.3.3.8] | ko:K00022 ko:K07516 ko:K10527 ko:K07514 ko:K01825 ko:K01782 | Fatty acid metabolism |
|  |  |  |  | ko00071_entry_62 | enoyl-CoA hydratase / long-chain 3-hydroxyacyl-CoA dehydrogenase [EC:4.2.1.17 1.1.1.211]; HADHA; enoyl-CoA hydratase/3-hydroxyacyl-CoA dehydrogenase [EC:4.2.1.17 1.1.1.35 1.1.1.211]; MFP2 | ko:K07515 ko:K10527 | Fatty acid metabolism |
|  |  |  |  | ko00071_entry_60 | enoyl-CoA hydratase / 3-hydroxyacyl-CoA dehydrogenase / 3,2-trans-enoyl-CoA isomerase [EC:4.2.1.17 1.1.1.35 5.3.3.8]; enoyl-CoA hydratase/3-hydroxyacyl-CoA dehydrogenase [EC:4.2.1.17 1.1.1.35 1.1.1.211]; ECHS1; HADHA; 3-hydroxyacyl-CoA dehydrogenase / enoyl-CoA hydratase / 3-hydroxybutyryl-CoA epimerase [EC:1.1.1.35 4.2.1.17 5.1.2.3]; ysiB, fadB; MFP2; fadB; enoyl-CoA hydratase / long-chain 3-hydroxyacyl-CoA dehydrogenase [EC:4.2.1.17 1.1.1.211]; fadJ; enoyl-CoA hydratase [EC:4.2.1.17]; paaF, echA; EHHADH; 3-hydroxyacyl-CoA dehydrogenase / enoyl-CoA hydratase / 3-hydroxybutyryl-CoA epimerase / enoyl-CoA isomerase [EC:1.1.1.35 4.2.1.17 5.1.2.3 5.3.3.8] | ko:K01692 ko:K10527 ko:K01825 ko:K01782 ko:K07511 ko:K13767 ko:K07514 ko:K07515 | Fatty acid metabolism |
|  |  |  |  | ko00071_entry_59 | HADH; 3-hydroxyacyl-CoA dehydrogenase [EC:1.1.1.35]; enoyl-CoA hydratase / 3-hydroxyacyl-CoA dehydrogenase / 3,2-trans-enoyl-CoA isomerase [EC:4.2.1.17 1.1.1.35 5.3.3.8]; enoyl-CoA hydratase/3-hydroxyacyl-CoA dehydrogenase [EC:4.2.1.17 1.1.1.35 1.1.1.211]; 3-hydroxyacyl-CoA dehydrogenase / enoyl-CoA hydratase / 3-hydroxybutyryl-CoA epimerase [EC:1.1.1.35 4.2.1.17 5.1.2.3]; fadN; fadB; fadJ; MFP2; EHHADH; 3-hydroxyacyl-CoA dehydrogenase / enoyl-CoA hydratase / 3-hydroxybutyryl-CoA epimerase / enoyl-CoA isomerase [EC:1.1.1.35 4.2.1.17 5.1.2.3 5.3.3.8] | ko:K00022 ko:K07516 ko:K10527 ko:K07514 ko:K01825 ko:K01782 | Fatty acid metabolism |
|  |  |  |  | ko00071_entry_55 | enoyl-CoA hydratase / long-chain 3-hydroxyacyl-CoA dehydrogenase [EC:4.2.1.17 1.1.1.211]; HADHA; enoyl-CoA hydratase/3-hydroxyacyl-CoA dehydrogenase [EC:4.2.1.17 1.1.1.35 1.1.1.211]; MFP2 | ko:K07515 ko:K10527 | Fatty acid metabolism |
|  |  |  |  | ko00071_entry_52 | enoyl-CoA hydratase / 3-hydroxyacyl-CoA dehydrogenase / 3,2-trans-enoyl-CoA isomerase [EC:4.2.1.17 1.1.1.35 5.3.3.8]; enoyl-CoA hydratase/3-hydroxyacyl-CoA dehydrogenase [EC:4.2.1.17 1.1.1.35 1.1.1.211]; ECHS1; HADHA; 3-hydroxyacyl-CoA dehydrogenase / enoyl-CoA hydratase / 3-hydroxybutyryl-CoA epimerase [EC:1.1.1.35 4.2.1.17 5.1.2.3]; ysiB, fadB; MFP2; fadB; enoyl-CoA hydratase / long-chain 3-hydroxyacyl-CoA dehydrogenase [EC:4.2.1.17 1.1.1.211]; fadJ; enoyl-CoA hydratase [EC:4.2.1.17]; paaF, echA; EHHADH; 3-hydroxyacyl-CoA dehydrogenase / enoyl-CoA hydratase / 3-hydroxybutyryl-CoA epimerase / enoyl-CoA isomerase [EC:1.1.1.35 4.2.1.17 5.1.2.3 5.3.3.8] | ko:K01692 ko:K10527 ko:K01825 ko:K01782 ko:K07511 ko:K13767 ko:K07514 ko:K07515 | Fatty acid metabolism |
|  |  |  |  | ko00071_entry_51 | HADH; 3-hydroxyacyl-CoA dehydrogenase [EC:1.1.1.35]; enoyl-CoA hydratase / 3-hydroxyacyl-CoA dehydrogenase / 3,2-trans-enoyl-CoA isomerase [EC:4.2.1.17 1.1.1.35 5.3.3.8]; enoyl-CoA hydratase/3-hydroxyacyl-CoA dehydrogenase [EC:4.2.1.17 1.1.1.35 1.1.1.211]; 3-hydroxyacyl-CoA dehydrogenase / enoyl-CoA hydratase / 3-hydroxybutyryl-CoA epimerase [EC:1.1.1.35 4.2.1.17 5.1.2.3]; fadN; fadB; fadJ; MFP2; EHHADH; 3-hydroxyacyl-CoA dehydrogenase / enoyl-CoA hydratase / 3-hydroxybutyryl-CoA epimerase / enoyl-CoA isomerase [EC:1.1.1.35 4.2.1.17 5.1.2.3 5.3.3.8] | ko:K00022 ko:K07516 ko:K10527 ko:K07514 ko:K01825 ko:K01782 | Fatty acid metabolism |
|  |  |  |  | ko00071_entry_43 | enoyl-CoA hydratase / 3-hydroxyacyl-CoA dehydrogenase / 3,2-trans-enoyl-CoA isomerase [EC:4.2.1.17 1.1.1.35 5.3.3.8]; enoyl-CoA hydratase/3-hydroxyacyl-CoA dehydrogenase [EC:4.2.1.17 1.1.1.35 1.1.1.211]; ECHS1; HADHA; 3-hydroxyacyl-CoA dehydrogenase / enoyl-CoA hydratase / 3-hydroxybutyryl-CoA epimerase [EC:1.1.1.35 4.2.1.17 5.1.2.3]; ysiB, fadB; MFP2; fadB; enoyl-CoA hydratase / long-chain 3-hydroxyacyl-CoA dehydrogenase [EC:4.2.1.17 1.1.1.211]; fadJ; enoyl-CoA hydratase [EC:4.2.1.17]; paaF, echA; EHHADH; 3-hydroxyacyl-CoA dehydrogenase / enoyl-CoA hydratase / 3-hydroxybutyryl-CoA epimerase / enoyl-CoA isomerase [EC:1.1.1.35 4.2.1.17 5.1.2.3 5.3.3.8] | ko:K01692 ko:K10527 ko:K01825 ko:K01782 ko:K07511 ko:K13767 ko:K07514 ko:K07515 | Fatty acid metabolism |
|  |  |  |  | ko00071_entry_42 | HADH; 3-hydroxyacyl-CoA dehydrogenase [EC:1.1.1.35]; enoyl-CoA hydratase / 3-hydroxyacyl-CoA dehydrogenase / 3,2-trans-enoyl-CoA isomerase [EC:4.2.1.17 1.1.1.35 5.3.3.8]; enoyl-CoA hydratase/3-hydroxyacyl-CoA dehydrogenase [EC:4.2.1.17 1.1.1.35 1.1.1.211]; 3-hydroxyacyl-CoA dehydrogenase / enoyl-CoA hydratase / 3-hydroxybutyryl-CoA epimerase [EC:1.1.1.35 4.2.1.17 5.1.2.3]; fadN; fadB; fadJ; MFP2; EHHADH; 3-hydroxyacyl-CoA dehydrogenase / enoyl-CoA hydratase / 3-hydroxybutyryl-CoA epimerase / enoyl-CoA isomerase [EC:1.1.1.35 4.2.1.17 5.1.2.3 5.3.3.8] | ko:K00022 ko:K07516 ko:K10527 ko:K07514 ko:K01825 ko:K01782 | Fatty acid metabolism |
|  |  |  |  | ko00071_entry_107 | enoyl-CoA hydratase / long-chain 3-hydroxyacyl-CoA dehydrogenase [EC:4.2.1.17 1.1.1.211]; HADHA; enoyl-CoA hydratase/3-hydroxyacyl-CoA dehydrogenase [EC:4.2.1.17 1.1.1.35 1.1.1.211]; MFP2 | ko:K07515 ko:K10527 | Fatty acid metabolism |
|  |  |  |  | ko00071_entry_104 | enoyl-CoA hydratase / 3-hydroxyacyl-CoA dehydrogenase / 3,2-trans-enoyl-CoA isomerase [EC:4.2.1.17 1.1.1.35 5.3.3.8]; enoyl-CoA hydratase/3-hydroxyacyl-CoA dehydrogenase [EC:4.2.1.17 1.1.1.35 1.1.1.211]; ECHS1; HADHA; 3-hydroxyacyl-CoA dehydrogenase / enoyl-CoA hydratase / 3-hydroxybutyryl-CoA epimerase [EC:1.1.1.35 4.2.1.17 5.1.2.3]; ysiB, fadB; MFP2; fadB; enoyl-CoA hydratase / long-chain 3-hydroxyacyl-CoA dehydrogenase [EC:4.2.1.17 1.1.1.211]; fadJ; enoyl-CoA hydratase [EC:4.2.1.17]; paaF, echA; EHHADH; 3-hydroxyacyl-CoA dehydrogenase / enoyl-CoA hydratase / 3-hydroxybutyryl-CoA epimerase / enoyl-CoA isomerase [EC:1.1.1.35 4.2.1.17 5.1.2.3 5.3.3.8] | ko:K01692 ko:K10527 ko:K01825 ko:K01782 ko:K07511 ko:K13767 ko:K07514 ko:K07515 | Fatty acid metabolism |
|  |  |  |  | ko00071_entry_103 | HADH; 3-hydroxyacyl-CoA dehydrogenase [EC:1.1.1.35]; enoyl-CoA hydratase / 3-hydroxyacyl-CoA dehydrogenase / 3,2-trans-enoyl-CoA isomerase [EC:4.2.1.17 1.1.1.35 5.3.3.8]; enoyl-CoA hydratase/3-hydroxyacyl-CoA dehydrogenase [EC:4.2.1.17 1.1.1.35 1.1.1.211]; 3-hydroxyacyl-CoA dehydrogenase / enoyl-CoA hydratase / 3-hydroxybutyryl-CoA epimerase [EC:1.1.1.35 4.2.1.17 5.1.2.3]; fadN; fadB; fadJ; MFP2; EHHADH; 3-hydroxyacyl-CoA dehydrogenase / enoyl-CoA hydratase / 3-hydroxybutyryl-CoA epimerase / enoyl-CoA isomerase [EC:1.1.1.35 4.2.1.17 5.1.2.3 5.3.3.8] | ko:K00022 ko:K07516 ko:K10527 ko:K07514 ko:K01825 ko:K01782 | Fatty acid metabolism |
| 51 | Endosperm | GRMZM2G368861_P01 | N6 |  |  |  |  |
| 52 | Endosperm | GRMZM2G019404_P01 | N5; N6 | ko00190_entry_107 | E3.6.3.6; H+-transporting ATPase [EC:3.6.3.6] | ko:K01535 | Oxidative phosphorylation |
| 53 | Endosperm | GRMZM2G041275_P01 | N4; N6 | ko00190_entry_105 | F-type H+-transporting ATPase subunit beta [EC:3.6.3.14]; ATPF0C, atpE; ATPVB, ntpB; ATPeF1D, ATP5D; V-type H+-transporting ATPase 16kDa proteolipid subunit [EC:3.6.3.14]; ATPVI, ntpI; ATPeF0F, ATP5J2; ATPVC, ntpC; F-type H+-transporting ATPase subunit f [EC:3.6.3.14]; F-type H+-transporting ATPase subunit gamma [EC:3.6.3.14]; ATPF1D, atpH; ATPeF1G, ATP5C1; ATPeFH, ATP14; ATPF1G, atpG; F-type H+-transporting ATPase subunit alpha [EC:3.6.3.14]; ATPeF0E, ATP5I; ATPeVE, ATP6E; F-type H+-transporting ATPase subunit a [EC:3.6.3.14]; V-type H+-transporting ATPase 54 kD subunit [EC:3.6.3.14]; ATPeF0B, ATP5F1; ATPF0B, atpF; ATPeF0A, MTATP6; ATPeFF, ATP17; ATPeF0F6, ATP5J; ATPF0A, atpB; V-type H+-transporting ATPase subunit E [EC:3.6.3.14]; F-type H+-transporting ATPase subunit g [EC:3.6.3.14]; V-type H+-transporting ATPase subunit A [EC:3.6.3.14]; ATPeV54kD; ATPeVPL, ATP6L; F-type H+-transporting ATPase subunit delta [EC:3.6.3.14]; F-type H+-transporting ATPase subunit 6 [EC:3.6.3.14]; V-type H+-transporting ATPase subunit B [EC:3.6.3.14]; F-type H+-transporting ATPase subunit d [EC:3.6.3.14]; ATPeVA, ATP6A1; ATPeVB, ATP6B1; ATPeFK, ATP19; F-type H+-transporting ATPase oligomycin sensitivity conferral protein [EC:3.6.3.14]; ATPVA, ntpA; V-type H+-transporting ATPase subunit H [EC:3.6.3.14]; ATPF1B, atpD; ATPeF1A, ATP5A1; V-type H+-transporting ATPase subunit I [EC:3.6.3.14]; V-type H+-transporting ATPase subunit D [EC:3.6.3.14]; F-type H+-transporting ATPase subunit k [EC:3.6.3.14]; V-type H+-transporting ATPase subunit AC39 [EC:3.6.3.14]; ATPeFJ, ATP18; ATPeVH, ATP6H; ATPeF1O, ATP5O; ATPeVS1, ATP6S1; ATPF1A, atpA; ATPVE, ntpE; ATPeVD, ATP6M; V-type H+-transporting ATPase subunit K [EC:3.6.3.14]; ATPeVG, ATP6G1; F-type H+-transporting ATPase subunit e [EC:3.6.3.14]; ATPF1E, atpC; F-type H+-transporting ATPase subunit j [EC:3.6.3.14]; ATPeF1E, ATP5E; ATPeF1B, ATP5B; ATPeF0C, ATP5G; ATPeF08, MTATP8; F-type H+-transporting ATPase subunit b [EC:3.6.3.14]; ATPVK, ntpK; ATPeVAC39, ATP6D; ATPeVC, ATP6C; ATPVF, ntpF; ATPVD, ntpD; V-type H+-transporting ATPase 21kDa proteolipid subunit [EC:3.6.3.14]; ATPeVF, ATP6S14; F-type H+-transporting ATPase subunit c [EC:3.6.3.14]; V-type H+-transporting ATPase S1 subunit [EC:3.6.3.14]; ATPeVI, ATP6N1A; ATPeFD, ATP5H; F-type H+-transporting ATPase subunit h [EC:3.6.3.14]; ATPeVPF, ATP6F; F-type H+-transporting ATPase subunit epsilon [EC:3.6.3.14]; V-type H+-transporting ATPase subunit G [EC:3.6.3.14]; ATPeFG, ATP5L; V-type H+-transporting ATPase subunit C [EC:3.6.3.14]; V-type H+-transporting ATPase subunit F [EC:3.6.3.14]; F-type H+-transporting ATPase subunit 8 [EC:3.6.3.14] | ko:K02108 ko:K02109 ko:K02110 ko:K02111 ko:K02112 ko:K02113 ko:K02114 ko:K02115 ko:K02117 ko:K02118 ko:K02119 ko:K02120 ko:K02121 ko:K02122 ko:K02123 ko:K02124 ko:K02125 ko:K02126 ko:K02127 ko:K02128 ko:K02129 ko:K02130 ko:K02131 ko:K02132 ko:K02133 ko:K02134 ko:K02135 ko:K02136 ko:K02137 ko:K02138 ko:K02139 ko:K02140 ko:K02141 ko:K02142 ko:K02143 ko:K02144 ko:K02145 ko:K02146 ko:K02147 ko:K02148 ko:K02149 ko:K02150 ko:K02151 ko:K02152 ko:K02153 ko:K02154 ko:K02155 ko:K03661 ko:K03662 | Oxidative phosphorylation |
| 54 | Endosperm | GRMZM2G325118_P01 | N6; N6-N4 |  |  |  |  |
| 55 | Endosperm | GRMZM5G876898_P01 | N5; N6 | ko00260_entry_114 | gcvT, AMT; aminomethyltransferase [EC:2.1.2.10] | ko:K00605 | Glycine, serine and threonine metabolism |
|  |  |  |  | ko00670_entry_42 | gcvT, AMT; aminomethyltransferase [EC:2.1.2.10] | ko:K00605 | One carbon pool by folate |
|  |  |  |  | ko00670_entry_64 | gcvT, AMT; aminomethyltransferase [EC:2.1.2.10] | ko:K00605 | One carbon pool by folate |
| 56 | Endosperm | GRMZM2G051764_P01 | N5; N6 |  |  |  |  |
| 57 | Endosperm | GRMZM2G019500_P01 | N4; N6 |  |  |  |  |
| 58 | Endosperm | GRMZM2G039263_P01 | N4; N6 |  |  |  |  |
| 59 | Endosperm | GRMZM2G038126_P01 | N4 |  |  |  |  |
| 60 | Endosperm | GRMZM2G059299_P01 | N4 |  |  |  |  |
| 61 | Endosperm | GRMZM2G025215_P01 | N4; N5 |  |  |  |  |
| 62 | Endosperm | GRMZM2G096475_P01 | N6; N6-N4 |  |  |  |  |
| 63 | Endosperm | GRMZM2G121137_P01 | N4; N5 |  |  |  |  |
| 64 | Endosperm | GRMZM2G025977_P01 | N4; N5 |  |  |  |  |
| 65 | Endosperm | GRMZM2G113332_P01 | N4; N5 |  |  |  |  |
| 66 | Endosperm | GRMZM2G075290_P01 | N4; N5; N6 |  |  |  |  |
| 67 | Endosperm | GRMZM2G119361_P01 | N4; N5; N6 |  |  |  |  |
| 68 | Endosperm | GRMZM2G358153_P01 | N5 |  |  |  |  |
| 69 | Endosperm | GRMZM2G304548_P01 | N5 |  |  |  |  |
| 70 | Endosperm | GRMZM2G009593_P01 | N4; N5; N6 |  |  |  |  |
| 71 | Endosperm | GRMZM2G098167_P01 | N4; N5; N6 |  |  |  |  |
| 72 | Endosperm | GRMZM2G051879_P01 | N4; N6 |  |  |  |  |
| 73 | Endosperm | GRMZM2G003595_P01 | N4; N5; N6 |  |  |  |  |
| 74 | Endosperm | GRMZM2G701221_P01 | N4; N5; N6 |  |  |  |  |
| 75 | Endosperm | GRMZM2G172357_P01 | N6 |  |  |  |  |
| 76 | Endosperm | GRMZM2G011523_P01 | N6 |  |  |  |  |
| 77 | Endosperm | GRMZM2G030902_P01 | N6 |  |  |  |  |
| 78 | Endosperm | GRMZM2G122357_P01 | N6 |  |  |  |  |
| 79 | Endosperm | GRMZM2G092474_P01 | N6 |  |  |  |  |
| 80 | Endosperm | GRMZM2G053206_P01 | N6 |  |  |  |  |
| 81 | Endosperm | GRMZM2G031572_P01 | N6 |  |  |  |  |
| 82 | Endosperm | AC225147.4_FGP002 | N6 |  |  |  |  |
| 83 | Endosperm | GRMZM2G084406_P02 | N6 |  |  |  |  |
| 84 | Endosperm | GRMZM2G111566_P01 | N5; N6 |  |  |  |  |
| 85 | Endosperm | GRMZM2G134176_P01 | N6 |  |  |  |  |
| 86 | Endosperm | GRMZM2G460860_P01 | N5; N6 |  |  |  |  |

**Table E.** Differentially expressed proteins identified by 2-DE.

| **Id** | **Protein** | **Protein/Domain** | **Function description** | **Function category** | USI | USEV |
| --- | --- | --- | --- | --- | --- | --- |
| 61 | gi|194701654 | YKR066c | Catalase (peroxidase I) | Inorganic ion transport and metabolism ; | 90.23 | 2.00E-112 |
| 73 | gi|226493460 | - | - | - | 39.19 | 4.00E-26 |
| 79 | gi|194703484* | all4902 | Glutathione S-transferase | Posttranslational modification, protein turnover, chaperones ; | 86.38 | 2.00E-116 |
| 84 | gi|226495167* | - | - | - | 51.13 | 9.00E-87 |
| 108 | gi|226528529 | SPAC29B12.04 | Pyridoxine biosynthesis enzyme | Coenzyme transport and metabolism ; | 92.16 | 1.00E-167 |
| 116 | gi|219363167 | all0438_2 | FOG: WD40 repeat | General function prediction only ; | 89.52 | 3.00E-171 |
| 172 | gi|226504732 | YJR159w | Threonine dehydrogenase and related Zn-dependent dehydrogenases | Amino acid transport and metabolism ; General function prediction only ; | 70.22 | 5.00E-157 |
| 229 | gi|3694807 | YLR089c | Aspartate/tyrosine/aromatic aminotransferase | Amino acid transport and metabolism ; | 93.98 | 0 |
| 230 | gi|162460735 | SPBC1815.01 | Enolase | Carbohydrate transport and metabolism ; | 100 | 0 |
| 273 | gi|226500876* | alr0577 | FKBP-type peptidyl-prolyl cis-trans isomerases 1 | Posttranslational modification, protein turnover, chaperones ; | 86.82 | 0 |
| 278 | gi|162463106 | AGl1564 | Phosphoglucomutase | Carbohydrate transport and metabolism ; | 100 | 0 |
| 315 | gi|31322756 | TM0272 | Phosphoenolpyruvate synthase/pyruvate phosphate dikinase | Carbohydrate transport and metabolism ; | 91.03 | 0 |
| 324 | gi|168586* | TM0272 | Phosphoenolpyruvate synthase/pyruvate phosphate dikinase | Carbohydrate transport and metabolism ; | 98.94 | 0 |
| 349 | gi|162458009* | YEL034w | Translation elongation factor P (EF-P)/translation initiation factor 5A (eIF-5A) | Translation, ribosomal structure and biogenesis ; | 100 | 2.00E-90 |
| 365 | gi|194688414 | ML0380 | Co-chaperonin GroES (HSP10) | Posttranslational modification, protein turnover, chaperones ; | 76.34 | 2.00E-94 |
| 397 | gi|162464321* | DR0325 | Malate/lactate dehydrogenases | Energy production and conversion ; | 100 | 0 |
| 445 | gi|23664291 | all4645 | ADP-glucose pyrophosphorylase | Carbohydrate transport and metabolism ; | 90.93 | 0 |
| 448 | gi|293331027* | YLR355c | Ketol-acid reductoisomerase | Amino acid transport and metabolism ; Coenzyme transport and metabolism ; | 97.28 | 0 |
| 454 | gi|293336560* | MA4007 | Phosphoglyceromutase | Carbohydrate transport and metabolism ; | 97.29 | 0 |
| 460 | gi|308081377* | SPAC13G7.02c | Molecular chaperone | Posttranslational modification, protein turnover, chaperones ; | 96.3 | 0 |
| 500 | gi|162460575 | PA3450 | Peroxiredoxin | Posttranslational modification, protein turnover, chaperones ; | 100 | 7.00E-134 |
| 697 | gi|195625630 | MJ0577 | Universal stress protein UspA and related nucleotide-binding proteins | Signal transduction mechanisms ; | 31.33 | 2.00E-06 |
| 795 | gi|194701304* | PAE0807 | 20S proteasome, alpha and beta subunits | Posttranslational modification, protein turnover, chaperones ; | 81.17 | 4.00E-108 |
| 797 | gi|226508518* | TM0555 | 3-isopropylmalate dehydratase small subunit | Amino acid transport and metabolism ; | 44.34 | 2.00E-13 |
| 812 | gi|223946111 | BU489 | Cold shock proteins | Transcription ; | 77.03 | 1.00E-25 |
| 899 | gi|195626012* | YKL150w | 2-polyprenylphenol hydroxylase and related flavodoxin oxidoreductases | Coenzyme transport and metabolism ; Energy production and conversion ; | 71.19 | 2.00E-124 |
| 920 | gi|195650645* | all0352 | Dehydrogenases with different specificities (related to short-chain alcohol dehydrogenases) | Lipid transport and metabolism ; Secondary metabolites biosynthesis, transport and catabolism ; General function prediction only ; | 46.73 | 1.00E-70 |
| 1053 | gi|194699136 | AGc447 | Predicted oxidoreductases (related to aryl-alcohol dehydrogenases) | Energy production and conversion ; | 70.15 | 3.00E-137 |
| 1133 | gi|194701098* | - | - | - | - | - |
| 1234 | gi|226503879 | aq_451 | Thiamine pyrophosphate-requiring enzymes [acetolactate synthase, pyruvate dehydrogenase (cytochrome), glyoxylate carboligase, phosphonopyruvate decarboxylase] | Amino acid transport and metabolism ; Coenzyme transport and metabolism ; | 99.82 | 0 |
| 2130 | gi|194705998 | SPCC24B10.21 | Triosephosphate isomerase | Carbohydrate transport and metabolism ; | 93.71 | 4.00E-73 |
| 2134 | gi|212275438* | YKL035w | UDP-glucose pyrophosphorylase | Carbohydrate transport and metabolism ; | 91.33 | 0 |
| 2137 | gi|226530305* | YKR066c | Catalase (peroxidase I) | Inorganic ion transport and metabolism ; | 91.2 | 2.00E-133 |
| 2139 | gi|157830250 | - | - | - | 100 | 2.00E-69 |
| 2142 | gi|219363213 | BS_citB | Aconitase A | Energy production and conversion ; | 93.58 | 0 |
| 2143 | gi|195610912 | - | - | - | - | - |

Note: * the proteins commonly identified by 2-DE and iTRAQ; USI Uniprot_Swissprot Identity, USEV Uniprot_Swissprot E_Value.

**Table F.** The functions and expression differences of inbred line N04 for endosperm proteins directly involved in regulating division, elongation and differentiation at 3 developmental stages

| **Function-Description** | **Protein Accession** | **Stage comparison** | | | | **Tissue comparison** | | | |
| --- | --- | --- | --- | --- | --- | --- | --- | --- | --- |
|  |  | **R(N2/N1)** | **R(N3/N2)** | **R(N5/N4)** | **R(N6/N5)** | | **R(N4/N1)** | **R(N5/N2)** | **R(N6/N3)** |
| Auxin-binding protein | GRMZM2G116204_P01 | - | - | ≈ | + | | ≈ | + | + |
| Auxin transport | GRMZM2G090542_P01 | + | ≈ | ≈ | ≈ | | - | - | - |
| Auxin transport | GRMZM2G169095_P01 | ≈ | ≈ |  | ≈ | | + | + | + |
| Auxin transport | GRMZM2G122135_P03 | ≈ | ≈ |  |  | | - |  | - |
| Auxin transport | GRMZM2G376731_P01 |  |  |  |  | | - |  |  |
| Gibberellin receptor GID1L2 | GRMZM2G079949_P01 | ≈ | ≈ |  |  | |  |  |  |
| Gibberellin receptor GID1L2 | GRMZM2G049675_P01 | ≈ | ≈ |  |  | | + |  | + |
| Gibberellin receptor GID1L2 | GRMZM5G898880_P01 | ≈ | ≈ | ≈ | + | |  | ≈ | + |
| Gibberellin 20 oxidase | GRMZM2G121700_P01 | ≈ |  | ≈ | ≈ | | - | - |  |
| Peroxiredoxin | GRMZM2G129761_P01 | + | + | + | ≈ | | ≈ | ≈ | - |
| Peroxiredoxin | GRMZM2G145449_P01 | + | - |  |  | |  |  |  |
| Peroxiredoxin | GRMZM2G055936_P01 | ≈ | - |  | - | | ≈ |  | + |
| Peroxiredoxin | GRMZM2G139680_P01 |  |  | ≈ | ≈ | | - |  |  |
| Peroxiredoxin | GRMZM2G036921_P01 | ≈ | - | ≈ | - | | + | + | + |
| Ascorbate peroxidase | GRMZM2G014397_P02 | ≈ | ≈ |  |  | |  |  |  |
| Ascorbate peroxidase | GRMZM2G054300_P01 |  | - | - | - | | + |  | ≈ |
| Ascorbate peroxidase | GRMZM2G140667_P01 | ≈ | - | ≈ | - | | - | - | - |
| Cell cycle control, cell division, chromosome partitioning ; | GRMZM2G031496_P01 | ≈ | ≈ |  |  | |  | ≈ | ≈ |
| Cell cycle control, cell division, chromosome partitioning ; | GRMZM5G881950_P01 |  |  | - | + | | - |  |  |
| Tubulins proteins | GRMZM2G066191_P01 | ≈ | - | ≈ | - | | + | + | + |
| Tubulins proteins | GRMZM2G172932_P01 | - | ≈ | ≈ | ≈ | | - | - | - |
| Tubulins proteins | GRMZM2G152466_P01 | - |  | - |  | | - | - |  |
| Tubulins proteins | AC234515.1_FGP003 | - |  |  |  | | - |  |  |
| Tubulins proteins | GRMZM2G071790_P01 | - | ≈ | + | - | | - | ≈ | - |
| Tubulins proteins | GRMZM2G042636_P01 | - | - | - |  | | - | - |  |
| Tubulins proteins | GRMZM2G083243_P01 | ≈ | - | - | ≈ | | - | - | ≈ |
| Tubulins proteins | GRMZM2G043822_P02 | ≈ | - | ≈ | - | | ≈ | ≈ | ≈ |
| Tubulins proteins | GRMZM2G051782_P01 |  |  |  |  | | ≈ |  |  |
| Tubulins proteins | GRMZM2G164696_P01 | ≈ | ≈ | ≈ | - | | - | - | - |
| Heat shock proteins | GRMZM2G083810_P01 |  |  | ≈ | + | | + |  |  |
| Heat shock proteins | GRMZM2G034157_P01 | + | + |  |  | |  |  |  |
| Heat shock proteins | AC208204.3_FGP006 | + | + | ≈ | ≈ | | + | + |  |
| Heat shock proteins | GRMZM2G404249_P01 |  |  |  |  | |  |  |  |
| Heat shock proteins | GRMZM2G080724_P01 |  |  | + | - | |  |  |  |
| Heat shock proteins | GRMZM2G007729_P02 | - | ≈ | + | - | | ≈ | + | ≈ |
| Heat shock proteins | GRMZM2G098167_P01 | + | + | + | ≈ | | + | + | + |
| Heat shock proteins | GRMZM2G044684_P01 |  |  | + | ≈ | | + |  |  |
| Heat shock proteins | GRMZM2G002440_P01 |  | + | ≈ | - | | + | + | + |
| Heat shock proteins | GRMZM5G833699_P01 |  |  | + | + | | ≈ |  |  |
| Heat shock proteins | GRMZM2G069651_P01 |  |  | ≈ | ≈ | | - |  |  |
| Heat shock proteins | GRMZM2G112165_P01 | - | - |  |  | | ≈ |  | + |
| Heat shock proteins | GRMZM2G002220_P01 |  |  | - | ≈ | | ≈ |  |  |
| Heat shock proteins | GRMZM2G012631_P01 | - |  | - | ≈ | | - | - | - |
| Heat shock proteins | GRMZM2G047434_P01 |  |  | ≈ | - | | ≈ |  |  |
| Heat shock proteins | GRMZM2G141931_P01 | ≈ | - |  |  | |  |  |  |
| Heat shock proteins | GRMZM2G039263_P01 |  |  |  |  | | - |  | - |
| Heat shock proteins | GRMZM2G069765_P01 | ≈ | ≈ | ≈ |  | | - | - | - |
| Heat shock proteins | GRMZM2G074790_P01 | ≈ | + |  |  | | - | - | - |
| Heat shock proteins | GRMZM2G109425_P01 | ≈ | ≈ |  |  | | - |  | - |
| Heat shock proteins | GRMZM2G083716_P01 | ≈ | + | - | + | |  | ≈ | + |
| Heat shock proteins | GRMZM2G381744_P01 | ≈ |  |  |  | | ≈ |  | ≈ |
| Heat shock proteins | AC215201.3_FGP005 |  |  | + | ≈ | | + |  |  |
| Heat shock proteins | GRMZM2G434173_P01 |  | + | ≈ | + | | - | - | - |
| Heat shock proteins | GRMZM2G111477_P01 | ≈ | + | ≈ | ≈ | | - | - | - |
| Heat shock proteins | EF517601.1_FGP012 | ≈ | ≈ |  |  | | - |  | - |
| Heat shock proteins | GRMZM2G416120_P01 | ≈ |  | ≈ | ≈ | | - | - | - |
| Heat shock proteins | GRMZM2G085909_P01 | - | ≈ |  |  | | - |  | - |
| Heat shock proteins | GRMZM2G015989_P01 | - | ≈ | ≈ | + | | - | - | ≈ |
| Heat shock proteins | GRMZM2G009871_P01 | ≈ | ≈ | ≈ | ≈ | | - | - | - |
| Heat shock proteins | GRMZM2G399284_P01 | ≈ | - | + | - | | + | + | + |
| Heat shock proteins | GRMZM2G127609_P01 | ≈ | - | + | - | | + | + | + |
| Heat shock proteins | GRMZM2G013652_P01 | - |  | + | - | | + | + |  |
| Heat shock proteins | GRMZM2G091189_P01 |  |  | ≈ | - | | + |  |  |
| Cysteine synthases | GRMZM2G005887_P01 |  | ≈ | - |  | | - | - | - |
| Cysteine synthases | GRMZM2G328893_P01 | ≈ | + |  |  | | + |  | + |
| Cysteine synthases | GRMZM2G006377_P01 | ≈ | + |  |  | |  |  |  |
| Cysteine synthases | GRMZM2G150256_P01 | - | + |  |  | |  |  |  |
| Cysteine synthases | GRMZM2G456217_P01 | + |  | ≈ | ≈ | | - | - |  |
| Cysteine synthases | GRMZM2G010435_P01 | + | + | ≈ | + | | - | - | - |
| Cysteine synthases | GRMZM2G073465_P03 | + | + | ≈ | + | | - | - | - |
| Cysteine synthases | GRMZM5G820822_P01 |  |  |  |  | |  |  |  |
| Actins | GRMZM2G126190_P01 | - | ≈ |  |  | |  |  |  |
| Actins | GRMZM2G126010_P01 | ≈ | + | - | + | | - | - | - |
| Actins | GRMZM2G030169_P01 | ≈ | ≈ | ≈ | - | | - | - | - |
| Actins | GRMZM2G067985_P04 | - | ≈ | ≈ | - | | - | ≈ | - |
| Actins | GRMZM2G047055_P01 | - | - | - | - | | + | + | + |
| Ras-related proteins | GRMZM2G173878_P01 | - | + |  |  | | + |  | + |
| Ras-related proteins | GRMZM2G061900_P01 |  |  | ≈ | - | | - |  | - |
| Ras-related proteins | GRMZM2G075719_P01 | ≈ | + | ≈ | - | | + | + | - |
| Ras-related proteins | GRMZM2G044368_P01 | ≈ | + | ≈ | - | | ≈ | ≈ | - |
| Ras-related proteins | GRMZM2G106960_P01 |  | - |  |  | |  |  |  |
| Pathogenesis-related protein | GRMZM2G075283_P01 | ≈ | + |  |  | |  |  |  |
| Pathogenesis-related protein | GRMZM2G465226_P01 | ≈ | + |  |  | |  |  |  |
| Pathogenesis-related protein | GRMZM2G481194_P01 | - | ≈ | ≈ | + | | + | + | + |
| Pathogenesis-related protein | GRMZM2G112524_P02 | + | - | ≈ | ≈ | | - | - | - |
| Pathogenesis-related protein | GRMZM2G102356_P01 | - |  | ≈ | - | | + | + |  |
| Dehydroascorbate reductase | GRMZM2G084881_P01 |  | ≈ | ≈ | ≈ | | + | ≈ | + |
| Dehydroascorbate reductase | GRMZM5G828229_P02 | ≈ | - |  |  | |  |  |  |
| Dehydroascorbate reductase | GRMZM2G134708_P01 | ≈ | ≈ |  |  | |  |  |  |
| Profilins, myosin and GDP dissociation inhibitor | GRMZM5G877388_P02 | ≈ | - |  |  | | - |  |  |
| Profilins, myosin and GDP dissociation inhibitor | GRMZM2G108780_P01 |  |  |  |  | | - |  |  |
| Glutathione S-transferase | GRMZM2G146246_P02 |  | ≈ | ≈ | - | | ≈ | - | - |
| Glutathione S-transferase | GRMZM2G134582_P01 | ≈ | ≈ | - | ≈ | | ≈ | - | - |
| Glutathione S-transferase | GRMZM2G330635_P01 |  |  | + | ≈ | | ≈ |  |  |
| Glutathione S-transferase | GRMZM5G855672_P02 | ≈ | - | + | - | |  | + | + |
| Glutathione S-transferase | GRMZM2G132093_P01 | ≈ | + | ≈ | ≈ | | ≈ | + | - |
| Glutathione S-transferase | GRMZM2G335618_P01 | ≈ | ≈ |  |  | |  |  |  |
| Glutathione S-transferase | GRMZM2G035502_P01 | ≈ |  | + | - | | ≈ | + |  |
| Glutathione S-transferase | GRMZM2G116273_P01 | ≈ | ≈ |  |  | | - |  | - |
| Glutathione S-transferase | GRMZM2G105005_P01 | ≈ | ≈ | ≈ | + | | ≈ | - | ≈ |
| Glutathione S-transferase | GRMZM2G122871_P01 | ≈ | ≈ |  |  | | + |  | ≈ |
| Glutathione S-transferase | GRMZM2G162486_P01 | ≈ | + | - | ≈ | | + | + | ≈ |
| Total | Up-expression + | 10 | 22 | 14 | 12 | | 24 | 19 | 19 |
|  | Down-expression - | 19 | 20 | 13 | 27 | | 39 | 26 | 32 |

**Table G.** The functions and expression differences of inbred line N04 for 45 endosperm proteins directly involved in starch biosynthesis and metabolism at 3 developmental stages

| **Function-Description** | **Protein Accession** | **Stage comparison** | | | | **Tissue comparison** | | | |
| --- | --- | --- | --- | --- | --- | --- | --- | --- | --- |
|  |  | **R(N2/N1)** | **R(N3/N2)** | **R(N5/N4)** | **R(N6/N5)** | | **R(N4/N1)** | **R(N5/N2)** | **R(N6/N3)** |
| ae (starch branching enzyme, SBEIIb) | GRMZM2G032628_P01 | - |  | - |  | |  |  |  |
| bt1 (ADP Glc) | GRMZM2G144081_P01 |  |  | + | - | |  |  |  |
| bt2 (glucose synthesis, AGPase SS) | GRMZM2G068506_P01 |  | + |  |  | | - |  |  |
| sbe1 (starch branching enzyme, SBEI) | GRMZM2G088753_P01 |  |  |  | + | | - |  |  |
| sbe3 (starch branching enzyme, SBEIII) | GRMZM2G073054_P01 | - | - |  |  | | + |  |  |
| sh1 (sucrose synthesis, SUS1) | GRMZM2G089713_P01 | - | + |  |  | |  |  |  |
| sh2 (glucose synthesis, AGPase LS) | GRMZM2G429899_P01 |  |  |  |  | | - |  |  |
| su1 (ISA1) | GRMZM2G138060_P01 |  |  |  |  | |  |  |  |
| wx1 (granule-bound starch synthase, GBSSI) | GRMZM2G024993_P01 |  | + |  | + | | - |  |  |
| zpu1 (starch debranching enzyme , PUL, DBE) | GRMZM2G158043_P02 | - | + |  | + | | - |  |  |
| Glucose synthesis | GRMZM2G004298_P01 |  | + |  |  | | - |  |  |
| Glucose synthesis | GRMZM2G027955_P01 | - |  |  | + | | + |  |  |
| Glucose synthesis | GRMZM2G065083_P01 |  |  |  |  | |  |  |  |
| Glucose synthesis | GRMZM2G106213_P01 |  |  | - | + | | + |  |  |
| Glucose synthesis | GRMZM2G140614_P01 |  |  |  | + | | + |  |  |
| Glucose synthesis | GRMZM2G163437_P01 |  |  | + |  | |  |  |  |
| Sucrose synthesis | GRMZM2G119689_P01 |  |  |  | - | | - |  |  |
| Sucrose synthesis | GRMZM2G139300_P01 |  | + |  | - | | + |  |  |
| Sucrose synthesis | GRMZM2G152908_P01 | + | - | - |  | | + |  |  |
| Sucrose synthesis | GRMZM2G318780_P02 | + | - |  | + | |  |  |  |
| Alpha-glucosidases, family 31 of glycosyl hydrolases | GRMZM2G428518_P01 | + | + |  | + | | - |  |  |
| Beta-glucosidase/6-phospho-beta-glucosidase/beta-galactosidase | GRMZM2G118003_P03 |  |  |  |  | | + |  |  |
| Beta-glucosidase-related glycosidases | GRMZM2G147687_P01 |  | + |  | + | | - |  |  |
| Endoglucanase | GRMZM2G147221_P01 |  |  | + |  | |  |  |  |
| Endopolygalacturonase | GRMZM5G831200_P01 |  |  |  | + | |  |  |  |
| Endopolygalacturonase | GRMZM2G079263_P01 |  | - |  | - | | - |  |  |
| Endopolygalacturonase | GRMZM2G119494_P01 |  | + | - | + | | - |  |  |
| Hexokinase | GRMZM2G104081_P01 |  |  |  |  | |  |  |  |
| Hexokinase | GRMZM2G104081_P01 |  |  |  |  | |  |  |  |
| Pectin methylesterase | GRMZM2G136106_P01 | + | + |  |  | |  |  |  |
| Pectin methylesterase | GRMZM2G167637_P01 |  | + |  | + | | - |  |  |
| Phosphoenolpyruvate synthase/pyruvate phosphate dikinase | GRMZM2G097457_P01 | + | - | - |  | | + |  |  |
| Phosphoglucomutase | GRMZM2G109383_P01 |  |  |  |  | | - |  |  |
| Phosphoglucomutase | GRMZM2G023289_P03 |  |  | - | + | | - |  |  |
| Predicted UDP-glucose 6-dehydrogenase | GRMZM5G862540_P01 |  |  |  |  | |  |  |  |
| Glucan phosphorylase | GRMZM2G074158_P01 |  | - | - | + | | - |  |  |
| Sugar kinases, ribokinase family | GRMZM2G086845_P01 |  | + |  |  | |  |  |  |
| Sugar kinases, ribokinase family | GRMZM2G051677_P01 |  | - | - | - | | - |  |  |
| Trehalose-6-phosphate synthase | GRMZM2G118462_P01 |  |  |  |  | |  |  |  |
| UDP-glucose pyrophosphorylase | GRMZM2G032003_P02 |  |  | - | + | | + |  |  |
| Function unknown | GRMZM2G097207_P01 |  |  |  | + | |  |  |  |
| Function unknown | GRMZM2G325008_P01 |  | + | - | + | |  |  |  |
| Function unknown | GRMZM2G458164_P01 |  | + |  |  | | + |  |  |
| Function unknown | GRMZM2G111143_P01 |  | + |  | + | |  |  |  |
| Function unknown | GRMZM2G058310_P01 |  |  |  |  | | + |  |  |
| Function unknown | GRMZM2G125032_P01 |  | + |  | + | |  |  |  |
|  | Up-expression + | 5 | 16 | 3 | 19 | | 11 | 0 | 0 |
|  | Down-expression - | 5 | 7 | 1 | 5 | | 16 | 0 | 0 |

**Table H**. The functions and expression differences of inbred line N04 for proteins directly related with the formation and structure of kernel pericarp

| **Function-Description** | **Protein Accession** | **Stage comparison** | | | | | **Tissue comparison** | | |
| --- | --- | --- | --- | --- | --- | --- | --- | --- | --- |
|  |  | **R(N2/N1)** | **R(N3/N2)** | **R(N5/N4)** | **R(N6/N5)** | **R(N4/N1)** | | **R(N5/N2)** | **R(N6/N3)** |
| Cytokinin-O-glucosyltransferase | GRMZM2G042865_P01 | ≈ | + |  |  |  | |  |  |
| Brassinosteroid insensitive | GRMZM2G339540_P04 | ≈ | + |  |  |  | |  |  |
| Gibberellin receptor | GRMZM2G079949_P01 | ≈ | ≈ |  |  |  | |  |  |
| Gibberellin receptor | GRMZM2G049675_P01 | ≈ | ≈ |  |  | + | |  | + |
| Gibberellin receptor | GRMZM5G898880_P01 | ≈ | ≈ | ≈ | + |  | | ≈ | + |
| Leucine-rich repeat extensin-like protein | GRMZM2G065292_P01 |  |  | - | ≈ | ≈ | |  |  |
| Leucine-rich repeat extensin-like protein | GRMZM2G021742_P01 | ≈ | + | + | - | - | | ≈ | - |
| Leucine-rich repeat extensin-like protein | GRMZM2G099295_P01 | - | + |  |  | - | |  | - |
| Leucine-rich repeat extensin-like protein | GRMZM2G082823_P01 | ≈ | + | + |  | + | | + |  |
| Leucine-rich repeat extensin-like protein | GRMZM5G805485_P01 | ≈ | ≈ |  |  | ≈ | |  |  |
| Alpha-xylosidase | GRMZM2G009282_P01 | ≈ | + |  |  |  | |  |  |
| Peroxiredoxin | GRMZM2G129761_P01 | + | + | + | ≈ | ≈ | | ≈ | - |
| Peroxiredoxin | GRMZM2G145449_P01 | + | - |  |  |  | |  |  |
| Peroxiredoxin | GRMZM2G055936_P01 | ≈ | - |  | - | ≈ | |  | + |
| Peroxiredoxin | GRMZM2G139680_P01 |  |  | ≈ | ≈ | - | |  |  |
| Peroxiredoxin | GRMZM2G036921_P01 | ≈ | - | ≈ | - | + | | + | + |
| Ascorbate peroxidase | GRMZM2G014397_P02 | ≈ | ≈ |  |  |  | |  |  |
| Ascorbate peroxidase | GRMZM2G054300_P01 |  | - | - | - | + | |  | ≈ |
| Ascorbate peroxidase | GRMZM2G140667_P01 | ≈ | - | ≈ | - | - | | - | - |
| Annexins | GRMZM2G061950_P01 | - | + |  |  |  | |  |  |
| Annexins | GRMZM2G064993_P01 | - | + |  |  |  | |  |  |
| Heat shock proteins | GRMZM2G021816_P01 | ≈ | - |  |  |  | |  |  |
| Tubulin | GRMZM2G143627_P01 | ≈ | - |  |  |  | |  |  |
| Dehydroascorbate reductases | GRMZM2G084881_P01 |  | ≈ | ≈ | ≈ | + | | ≈ | + |
| Dehydroascorbate reductases | GRMZM5G828229_P02 | ≈ | - |  |  |  | |  |  |
| Dehydroascorbate reductases | GRMZM2G134708_P01 | ≈ | ≈ |  |  |  | |  |  |
| Pathogenesis-related protein | GRMZM2G075283_P01 | ≈ | + |  |  |  | |  |  |
| Pathogenesis-related protein | GRMZM2G112524_P02 | + | - | ≈ | ≈ | - | | - | - |
| Pathogenesis-related protein | GRMZM2G102356_P01 | - |  | ≈ | - | + | | + |  |
| Pathogenesis-related protein | GRMZM2G465226_P01 | ≈ | + |  |  |  | |  |  |
| Actin | GRMZM2G126190_P01 | - | ≈ |  |  |  | |  |  |
| Ras-related protein | GRMZM2G106960_P01 |  | - |  |  |  | |  |  |
| Glutathione S-transferase | GRMZM2G335618_P01 | ≈ | ≈ |  |  |  | |  |  |
| Late stage and putative 26S proteasome | GRMZM5G821637_P05 | - | ≈ |  |  |  | |  |  |
| Total | Up-expression + | 3 | 11 | 3 | 1 | 6 | | 3 | 5 |
|  | Down-expression - | 6 | 10 | 2 | 6 | 5 | | 2 | 5 |
